# Spatial and temporal evolution of distal 10q deletion, a prognostically unfavorable event in diffuse low-grade gliomas

**DOI:** 10.1186/s13059-014-0471-6

**Published:** 2014-09-23

**Authors:** Hinke F van Thuijl, Ilari Scheinin, Daoud Sie, Agusti Alentorn, Hendrik F van Essen, Martijn Cordes, Ruth Fleischeuer, Anja M Gijtenbeek, Guus Beute, Wimar A van den Brink, Gerrit A Meijer, Miek Havenith, Ahmed Idbaih, Khê Hoang-Xuan, Karima Mokhtari, Roel GW Verhaak, Paul van der Valk, Mark A van de Wiel, Jan J Heimans, Eleonora Aronica, Jaap C Reijneveld, Pieter Wesseling, Bauke Ylstra

**Affiliations:** Department of Pathology, VU University Medical Center, 1007 MB Amsterdam, The Netherlands; Department of Neurology, VU University Medical Center, 1007 MB Amsterdam, The Netherlands; Department of Pathology, Haartman Institute and HUSLAB, University of Helsinki and Helsinki University Central Hospital, 00014 Helsinki, Finland; Université Pierre et Marie Curie-Paris 6 Centre de Recherche de l’Institut du Cerveau et de la Moelle Epinière (CRICM), 75013 Paris, France; Inserm U975, 75013 Paris, France; Centre National de la Recherche Scientifique (CNRS), 75013 Paris, France; AP-HP, Groupe Hospitalier Pitié-Salpêtrière, Department of Neurology 2-Mazarin, 75013 Paris, France; Department of Pathology, Elisabeth Hospital Tilburg, 5022 GC Tilburg, The Netherlands; Department of Neurology, Radboud University Medical Center, 6525 GANijmegen, The Netherlands; Department of Neurological Surgery, Elisabeth Hospital Tilburg, 5022 GC Tilburg, The Netherlands; Department of Neurological Surgery, Isala, 8011 JW Zwolle The Netherlands; Department of Pathology, Isala, 8011 JW Zwolle The Netherlands; AP-HP, Groupe Hospitalier Pitié-Salpêtrière, Department of Neuropathology, 2-Mazarin, 75013 Paris, France; Departments of Genomic Medicine, University of Texas, MD Anderson Cancer Center, Houston, TX 77054 USA; Department of Bioinformatics and Computational Biology, University of Texas, MD Anderson Cancer Center, Houston, TX 77230 USA; Department of Epidemiology and Biostatistics, VU University Medical Center, 1007 MB Amsterdam, The Netherlands; Department of Mathematics, VU University, 1081 HV Amsterdam, The Netherlands; Department of Pathology, Academical Medical Center, 1105 AZ Amsterdam, The Netherlands; Department of Neurology, Academical Medical Center, 1105 AZ Amsterdam, The Netherlands; Department of Pathology, Radboud University Medical Center, 6525 GA Nijmegen, The Netherlands

## Abstract

**Background:**

The disease course of patients with diffuse low-grade glioma is notoriously unpredictable. Temporal and spatially distinct samples may provide insight into the evolution of clinically relevant copy number aberrations (CNAs). The purpose of this study is to identify CNAs that are indicative of aggressive tumor behavior and can thereby complement the prognostically favorable 1p/19q co-deletion.

**Results:**

Genome-wide, 50 base pair single-end sequencing was performed to detect CNAs in a clinically well-characterized cohort of 98 formalin-fixed paraffin-embedded low-grade gliomas. CNAs are correlated with overall survival as an endpoint. Seventy-five additional samples from spatially distinct regions and paired recurrent tumors of the discovery cohort were analyzed to interrogate the intratumoral heterogeneity and spatial evolution. Loss of 10q25.2-qter is a frequent subclonal event and significantly correlates with an unfavorable prognosis. A significant correlation is furthermore observed in a validation set of 126 and confirmation set of 184 patients. Loss of 10q25.2-qter arises in a longitudinal manner in paired recurrent tumor specimens, whereas the prognostically favorable 1p/19q co-deletion is the only CNA that is stable across spatial regions and recurrent tumors.

**Conclusions:**

CNAs in low-grade gliomas display extensive intratumoral heterogeneity. Distal loss of 10q is a late onset event and a marker for reduced overall survival in low-grade glioma patients. Intratumoral heterogeneity and higher frequencies of distal 10q loss in recurrences suggest this event is involved in outgrowth to the recurrent tumor.

**Electronic supplementary material:**

The online version of this article (doi:10.1186/s13059-014-0471-6) contains supplementary material, which is available to authorized users.

## Background

Diffuse low-grade gliomas (LGGs) are regarded as slow growing malignant brain tumors and patients can live up to 30 years with this disease. In a subset of patients the tumor exerts a more aggressive behavior and survival can be as short as two years [1]. Personalized timing of postoperative treatment is crucial to forestall progression in the latter group whilst preventing long-term side-effects for patients with more favorable prospects [2]. The disease course of patients with LGGs is correlated with gene mutations, such as in *p53* and *IDH1*, hypermethylation of *MGMT* as well as chromosomal copy number aberrations (CNAs). Regarding the latter, assessment of combined loss of 1p and 19q currently is implemented in routine clinical care in specific glioma subgroups given its favorable prognostic and predictive value [3,4]. Other CNAs, such as losses of chromosomes 10 and 11p, have been reported to be prognostically unfavorable, but have not been introduced into clinical practice yet, possibly due the limited number of samples included in the studies and/or lack of validation [5-7]. Unfavorable events might go undetected as a consequence of intratumoral heterogeneity in gliomas [8], which is particularly salient if they are only present in the more malignant subclones of LGGs that seed outgrowth of a recurrent tumor [9], thereby promoting a large extent of resection. As current knowledge on the temporal and spatial evolution of CNAs in LGGs is limited, we evaluated CNAs in a clinically and histologically representative cohort of formalin-fixed archival samples using shallow whole genome sequencing (shallow WGS). We demonstrate that loss of part or whole chromosome 10q is prognostically unfavorable and often present in a subclonal manner.

## Results

### Clinical and histological data

**Figure 1:**
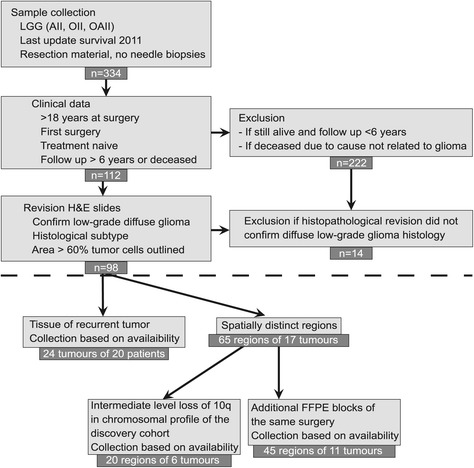
**Accrual of samples and clinical data of the discovery cohort.** Samples were selected based on criteria listed in the boxes connected with vertical arrows. Reasons for exclusion of samples are listed in boxes in the right panel, connected with horizontal arrows. The number below each box represents the number of patients. Boxes below the dotted line list criteria for collection of recurrent tumors and spatially distinct regions of LGGs. AII, astrocytoma; H&E, hematoxylin and eosin; OII, oligodendroglioma; OAII, oligoastrocytoma.

We studied 173 formalin-fixed paraffin-embedded (FFPE) samples from 98 LGG patients, including spatially distinct regions and paired recurrent tumors, by shallow WGS. Patients had either deceased or had passed the median survival time of six years for LGGs. Other inclusion criteria and patient characteristics of this discovery cohort are summarized in Figure 1 and Table 1. Age at diagnosis, overall survival and postoperative treatment (type and timing) varied extensively between patients, but not between the five participating hospitals, which contributed nearly equal numbers of cases. Comparison of overall survival between patients treated immediately after surgery and those for whom postoperative treatment was withheld did not reveal statistically significant differences. Characteristics of the LGG patients of the French validation (n =126) [5] and confirmation cohorts (n =184), the latter from publicly available data from The Cancer Genome Atlas (TCGA) [10], are also listed in Table 1. Due to the retrospective character of this study, the cohorts are not matched; there is considerable variation in duration of follow-up and the percentage of patients deceased and for which information on overall survival is available, as well as in the distribution of histological subgroups.

**Table 1:** **Characteristics of diffuse low-grade glioma patients in the discovery, validation and confirmation cohorts**

**Variable**	**Dutch discovery cohort (n =98)**	**French validation cohort (n =126)**	**TCGA confirmation cohort (n =184)**
Gender			
Female	50 (51%)	59 (47%)	93 (51%)
Age at diagnosis (years)			
Mean	40.3	39.8	40.8
Median	39.6	39.4	39
Range	(21-83)	(18-76)	(14-87)
Duration of follow-up of patients still alive at last evaluation (months)			
Mean	133.6	39.6	23.7
Median	129.0	30.9	10.7
Range	72-288	1-187	1-185
Patients deceased	46 (47%)	32 (25%)	18 (10%)
Overall survival of patients deceased at last evaluation (months)			
Mean	89	48.6	63.5
Median	149.0	51.8	65.6
Range	1-361	0.1-98	1.2-132.6
Histological subtypes			
Oligodendroglioma	43 (43%)	53 (42%)	87 (47%)
Astrocytoma	42 (42%)	23 (18%)	40 (22%)
Oligoastrocytoma	15 (15%)	50 (40%)	57 (31%)

### Copy number detection by shallow WGS in LGGs

**Figure 2:**
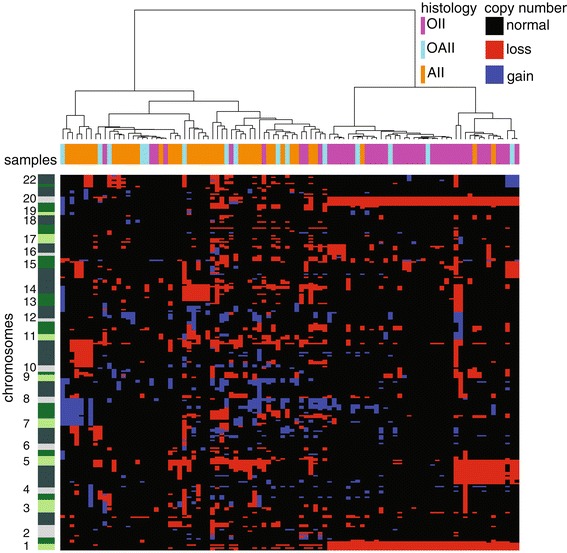
**Unsupervised clustering of CNAs in the discovery cohort.** Histological subtypes and patients are color-coded on the x-axis and chromosomes are ordered on the y-axis, 1 to 22 from bottom to top. Shades of green enable visualization of individual chromosomal arms, their size varying by the number of regions. Hence, a chromosomal arm with many breakpoints based on CNAs is depicted as larger compared with one with fewer breakpoints. Red, copy number loss; blue, copy number gain; black, no CNA. OII, oligodendroglioma; OAII, oligoastrocytoma; AII, astrocytoma.

To obtain genome-wide copy numbers from the FFPE samples of our discovery cohort, we evaluated the use of shallow WGS. First, for sample LGG284 a paired-end 100 (PE100) sequence run was performed. Copy number profiles were produced by counting the unique sequence tags per 15 kb bin of the paired-end 100 bp reads from both ends (PE100 in Figure S1A in Additional file 1), the single 100 bp read from one end (SR100 in Figure S1B in Additional file 1) and the trimmed first single 50 bp read from the same end (SR50 in Figure S1C in Additional file 1). The noise (measured as variance) of the different profiles is very similar and CNAs observed are indistinguishable from each other, which implies that the uniqueness of the 50 bp sequence tags suffices to infer copy number levels, and longer reads are not necessary. Array comparative genomic hybridization (array CGH) was performed on the same DNA sample, which confirmed the CNAs detected (Figure S1D in Additional file 1). For an additional eight samples both 50 bp single-read (SR50) shallow WGS and array CGH were applied as technical validation. Shallow WGS and array analysis invariably yielded the same CNA profiles (Figure S2 in Additional file 1). Based on this information, all subsequent analyses were performed using 50 bp single-read (SR50) shallow WGS since it is more cost-effective and allows the use of samples with short DNA fragments, which are frequently obtained with FFPE materials. The most frequent CNAs, detected in more than 10% of cases, are whole or partial loss of chromosomal arms 9p, 10q, 12p, 13 and 14, as well as gain of chromosomal arms 7q, 8q, 10p and 11q. The most frequent CNAs in this cohort are co-deletion of 1p and 19q often accompanied by loss of whole chromosome 4, all commensurate with previous reports [11] (Figure 2).

### The prognostic value of CNAs in discovery, validation and confirmation cohorts

**Figure 3:**
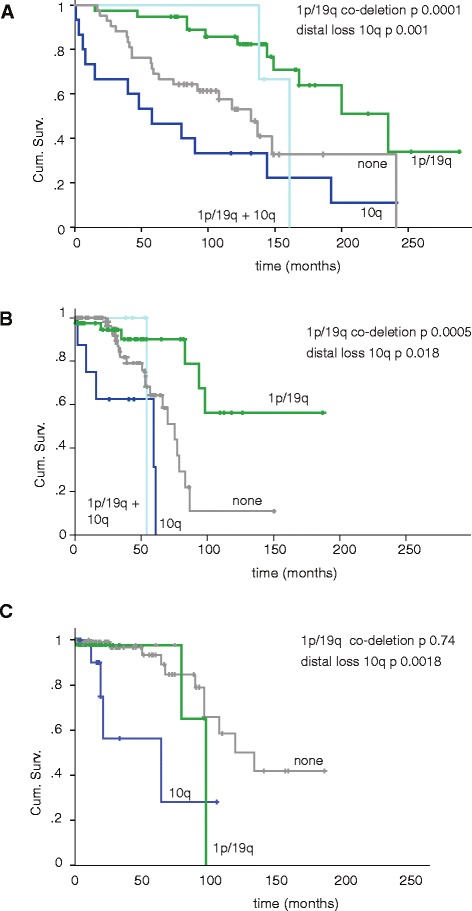
**Kaplan Meier plots for distal 10q loss and 1p/19q co-deletion in (A) discovery, (B) validation and (C) confirmation cohorts.** The dark blue line indicates loss of distal 10q without 1p/19q co-deletion versus the rest of the cohort (n =15, *P*-value =0.001 in **(A)**, n =8, *P*-value =0.018 in **(B)**, and n =14, *P*-value =0.0018 in **(C)**. The green line indicates 1p/19q co-deletion without distal loss of 10q (n =38, *P*-value =0.0001 in **(A)**, n =41, *P*-value =0.0005 in **(B)**, and n =47, *P*-value =0.74 in **(C)**. The light blue line indicates 10q loss and 1p/19q co-deletion (n =3, *P*-value =0.39 in **(A)**, n =4, *P*-value =0.94 in **(B)**, and n =0 in **(C)**. The grey line indicates neither 10q deletion nor 1p/19q co-deletion (n =42 in **(A)**, n =73 in **(B)**, and n =123 in **(C)**. The y-axis represents the fraction of patients alive, cumulative survival (Cum. Surv.), and the x-axis time in months. Censored patients are indicated with a vertical bar.

Association of survival with CNAs detected in the discovery cohort was tested. In addition to the known prognostically favorable 1p/19q co-deletion, five further chromosomal losses at chromosomes 9p, distal 10q, 11p, 13q, and 22q presented with statistical significance (Table 2). No associations were observed with gains. Significant regions were verified in the French validation cohort of 126 diffuse LGG patients (Table 1). Loss of distal 10q was an unfavorable CNA in both cohorts, whereas losses of chromosomal regions at 9p, 11p, 13q and 22q were not substantiated in the validation cohort (Table 2). In the discovery cohort, median overall survival for patients with or without loss of whole or distal 10q was respectively 6.6 years versus 16.7 years (18/98, *P*-value =0.009). The size of chromosome 10 deletion varies from whole chromosome loss (5/18) to 22.5 Mbp distal loss (10q25.2- 10qter). An association between loss of this region with overall survival was finally tested in the TCGA dataset of LGG. Despite the limited number of patients deceased in this cohort (Table 1), a significant association with overall survival was observed (*P*-value =0.0018) (Figure 3), which confirms that distal 10q is a prognostically unfavorable chromosomal aberration.

**Table 2:** **Prognostically unfavorable chromosomal regions of loss in diffuse low-grade gliomas**

**Chromosome**	**Start**	**End**	**Cytoband**	**Discovery cohort (%)**	**Validation cohort (%)**	**Discovery cohort**	**Validation cohort**
						**(** ***P*** **-value)**	**(** ***P*** **-value)**
9	24450001	28650000	9p21.3-21.1	21	7	0.039	1
10	112950001	135435000	10q25.2-qter	18	10	0.009	0.041
11	195001	14250000	11p15.5-15.2	13	15	0.0006	1
13	19500001	92550000	13q12.1-31.3	17	13	0.0001	1
22	34350001	51180000	22q12.3-13.33	11	8	0.000004	1

Frequency and *P*-value in discovery and validation cohorts calculated by log rank test and adjusted for multiple testing by Benjamini Hochberg and Holm Bonferroni, respectively. Positions according to GRCh37/hg19.

In the discovery cohort, absence of *IDH1* or *IDH2* mutation (11/98) was overrepresented in patients with distal 10q loss (7/11; five with whole chromosome 10 loss and two with distal 10q loss). After splitting the cohort by *IDH* status, a trend for distal 10q loss was observed; in the *IDH* mutant subgroup (n =87) the log rank test for loss of 10q (n =11) yielded a *P*-value of 0.077, and in the *IDH* wild-type subgroup (n =11), a similar *P*-value of 0.068 was yielded through the test for distal loss of 10q (n =7).

Co-deletion of 1p/19q was predominantly detected in LGGs with oligodendroglial histological features while loss of distal 10q was more frequently identified in astrocytic LGGs. However, there was no one-to-one relationship between histological features and these CNAs (Figure 2). Co-deletion of 1p/19q combined with distal 10q loss was observed in three LGGs of the discovery cohort and four LGGs of the validation cohort (all with oligodendroglial features) and none in the confirmation cohort. This limited number of patients does not allow for proper statistical survival analysis, but median survival of the patients in these cohorts combined (13.4 years) suggests that loss of 1p/19q and distal 10q counteracts overall survival. Simultaneous testing of both CNAs classified LGG patients with a favorable (1p/19q co-deletion), unfavorable (distal 10q loss), or intermediate (both) prognosis in all three cohorts (Figure 3A,B,C). In the discovery cohort, hazard ratios of 1p/19q co-deletion without distal 10q loss and of distal 10q loss without 1p/19q co-deletion were 0.30 (95% confidence interval 0.15 to 0.58), and 2.91 (95% confidence interval 1.53 to 5.55), respectively (Table 3).

**Table 3:** **Association of clinical and genetic parameters with overall survival in the discovery cohort**

**Parameter**	**n**	***P*** **-value**	**HR**
Age >50 years	23/98	0.187	1.57 (0.80-3.07)
Pre-operative KPS score <80	5/83	0.101	2.42 (0.79-7.05)
Pre-operative use of steroids	18/85	0.068	1.84 (0.99-3.58)
Pre-operative mass effect	37/71	0.0008	3.31 (1.61-6.73)
Pre-operative enhancement	35/79	0.031	2.16 (1.05-4.43)
Partial resection	69/90	0.029	2.85 (1.19-7.47)
Oligodendroglial histology	42/98	0.016	0.47 (0.25-0.87)
*IDH1* or *IDH2* mutation	86/97	0.071	0.47 (0.21-1.07)
1p/19q co-deletion without 10q loss	41/98	0.0001	0.30 (0.15-0.58)
Loss of 10q without 1p/19q co-deletion	15/98	0.001	2.91 (1.53-5.55)

Results were determined using a log rank test. n, patients in subgroup compared with total number of patients with available data for each parameter; HR, hazard ratio (95% confidence interval). KPS, Karnofsky Performance Score.

### Intratumoral heterogeneity of CNAs in LGGs

**Figure 4:**
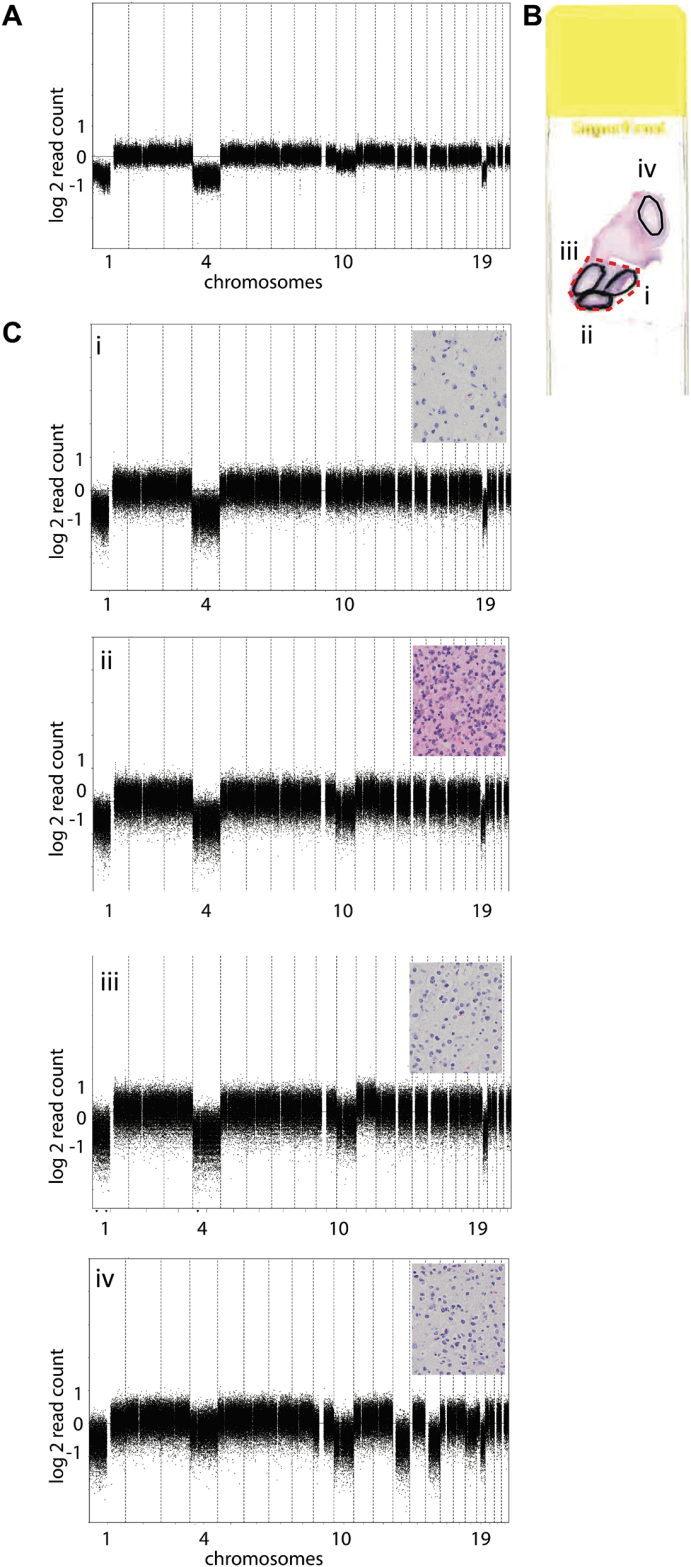
**Chromosomal copy number profiles for sample 240 demonstrating intratumoral copy number heterogeneity. (A)** CNA profile of initial tumor, clonal 1p/19q co-deletion, loss of chromosome 4 and intermediate level of loss of chromosome 10. **(B)** Hematoxylin and eosin stained slide showing regions used for DNA isolation: the red dotted line corresponds to the region used for chromosomal profile of 4A and regions outlined with a solid black line (labeled i to iv) were used for the chromosomal profiles of 4C. **(C)** CNA profiles from four non-overlapping regions. Insets at the top right corner of each profile show histological features representative for individual regions. In all regions the histopathological diagnosis was LGG, although within a tumor the regions analyzed for spatial heterogeneity often showed some variation in microscopic features, such as cellularity and nuclear size and shape. (i) Clonal 1p/19q co-deletion and loss of chromosome 4; (ii) clonal 1p/19q co-deletion, loss of chromosome 4 and intermediate loss of chromosome 10; (iii) clonal 1p/19q co-deletion, loss of chromosome 4, intermediate loss of chromosome 10 and intermediate gain of chromosome 11; (iv) clonal 1p/19q co-deletion, intermediate loss of chromosomes 4, 10q, 13, 15 and 18. The y-axis represents normalized log2 sequence read counts per bin, and the x-axis represents 15 kb bins ordered by genomic position from chromosomes 1 to 22.

In addition to the above-mentioned CNAs detected in single samples, we studied intratumoral heterogeneity by shallow WGS of multiple, spatially distinct regions obtained during the same surgery. Among other CNAs, distribution of 10q loss was assessed, illustrated for LGG240 in Figure 4 (more examples are provided in Additional file 2). In the original chromosomal profile of LGG240, marginal deflection of 10q was observed (a smaller distance from the 0-line than for the losses in 1p, 4, or 19q (Figure 4A) [12]. Assuming clonality of the 1p/19q co-deletion [13], this difference in extent of copy number loss suggests that 10q loss would only be present in about 30 to 35% of the tumor cells (Figure S3 in Additional file 1).

**Figure 5:**
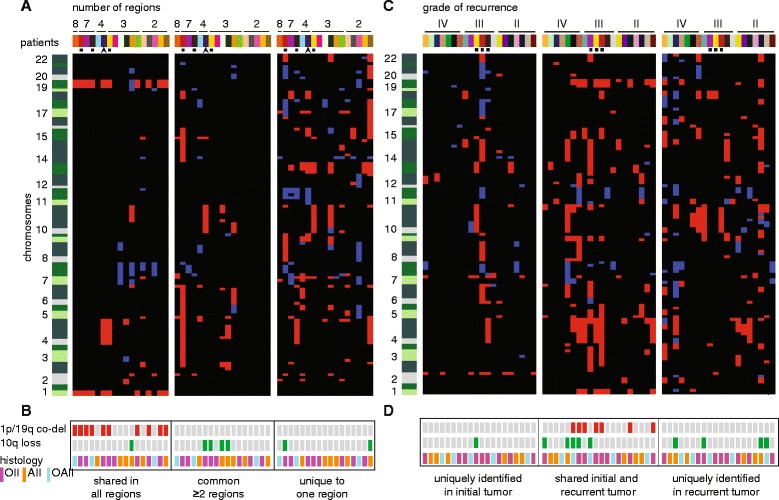
**Spatial and temporal evolution of CNAs in LGGs and paired recurrent tumors. (A)** CNAs in spatially distinct regions of LGGs of 17 patients. CNAs are categorized by detection in all regions (left panel), more than one region but not all regions (middle), or one region (right). Patients are ordered by the number of regions analyzed of each LGG from high to low. **(B)** Summary of prognostically relevant CNAs in spatially distinct regions and histology. No intratumoral heterogeneity was observed for 1p/19q co-deletion in any of the tumors, while distal 10q loss was often only detected in subclones. OII, oligodendroglioma; AII, astrocytoma; OAII, oligoastrocytoma. **(C)** CNAs in initial and paired recurrent tumors of 20 patients. CNAs are categorized by detection in initial tumor only (left panel), both initial and recurrence (middle) or detection uniquely in the recurrence (right). Patients are ordered by the histological malignancy grade of the recurrent tumor. **(D)** Summary of prognostically relevant CNAs in paired initial and recurrent tumors. 1p/19q co-deletion is stable over time, while distal 10q loss surfaces in recurrences, including two with a higher malignancy grade than LGG. Patients are color-coded on the x-axis and chromosomes are ordered on the y-axis, 1 to 22 from bottom to top. Shades of green enable visualization of individual chromosomal arms, their size varying by the number of regions. Hence, a chromosomal arm with many breakpoints based on CNAs is depicted as larger compared with one with fewer breakpoints. CNAs smaller than 5 Mbp were excluded from this figure. Red, copy number loss; blue, copy number gain; black, no CNA. The arrowhead indicates patient 240, the black squares three LGGs analyzed for both spatial and temporal evolution.

To further delineate intratumoral heterogeneity of CNAs in this sample, the originally outlined area was divided into three sub-regions and an additional tumor region within the same paraffin section was included (Figure 4B). The 1p/19q co-deletion as well as chromosome 4 loss were present in all sub-regions and assumed to be clonally present. Losses of chromosomes 9, 10, 13, 15, 18 and gain of chromosome 11 were present in one or few sub-regions and assumed to be heterogeneously present (Figure 4C). To technically validate the intratumoral copy number heterogeneity observed in LGG240, array CGH was performed for all but one (insufficient amount of DNA) of the spatially distinct regions, which confirmed either clonality of 1p/19q and chromosome 4 losses or heterogeneity of all six chromosomally aberrant regions (Figure S4 in Additional file 1). Three additional samples with a clonal type of deflection and four with a marginal deflection of (distal) 10q were technically validated by array CGH (Figure S2 in Additional file 1). Intratumoral heterogeneity was detected in 15 out of 17 LGGs analyzed for this purpose; 68% of the CNAs (84/124) were not homogeneously present in spatially distinct regions obtained during the same surgery, such as loss of chromosomal arm 5q, chromosome 13 and gain of 11p (Figure 5A). Co-deletion of 1p and 19q was the only CNA that was consistently present in all spatially distinct regions of LGGs with this combination of CNAs; others, such as gain of chromosomal arm 7q, were most often, but not always, clonal. Loss of 10q was heterogeneously present in seven out of eight patients (Figure 5B). Histological variability did not correspond to the extent of heterogeneity.

### Temporal evolution of CNAs in LGGs

**Figure 6:**
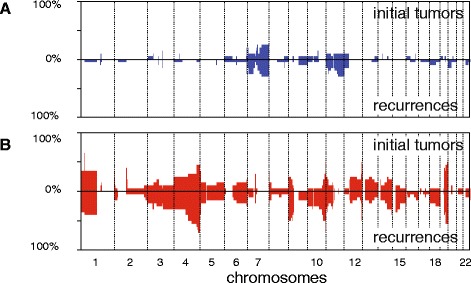
**CNAs in initial and recurrent tumors. (A)** Gains; **(B)** losses. The top of each graph shows the initial tumors, and the bottom the recurrences. Partial loss of chromosomal arm 4q, 9p and 10q were more frequently detected in recurrences. Bins are ordered by genomic position and from chromosomes 1 to 22 on the x-axis; percentages of cases showing CNAs are depicted on the y-axis.

Forty-seven out of 98 patients were subjected to a second surgery because of tumor progression. Of 20 patients, 24 recurrent tumors could be retrieved from medical archives. Almost 50% of CNAs (99/207) in the initial and paired recurrent tumors were shared and 15% (31/207) were uniquely detected in the initial tumor. A substantial proportion (37%, 77/207) of CNAs was uniquely identified in the recurrent tumor, such as loss of genomic regions at chromosomes 4, 10 and 15 (Figures 5C and 6). 1p/19q co-deletion was consistently identified in initial as well as recurrent tumors and there were no cases with new 1p/19q co-deletion. In four patients, *de novo* loss of 10q (including distal 10q losses) surfaced in the recurrence. In hindsight, marginal deflection of 10q was observed in the initial tumor of one of these four patients, and was not detected by the calling algorithm [12]. In two out of four patients with new loss of 10q a higher malignancy grade (WHO grade III or IV) had been assigned to the recurrent tumor (Figure 5D). In one of the three patients for which both spatially distinct regions of the initial tumor and recurrences were analyzed, subclonal 10q loss was present in one of the regions of the initial tumor, but undetectable in the recurrence (Additional file 3). In the other two patients, 10q loss was detected in both the initial and paired recurrent tumor.

## Discussion

Intratumoral heterogeneity at the genomic level has been observed in numerous types of cancer, although its implications for treatment often remain undetermined. In the present study, archival LGG material and matched clinical data provide insight into spatial and temporal evolution of prognostically relevant CNAs. All LGG samples collected for this study were included for copy number profiling by shallow WGS combined with a depth of coverage approach, yielding high quality data without technical dropouts. This approach proved to be particularly beneficial for our study, since no matched normal DNA is required, which is a major advantage when analyzing long-term archived FFPE tumor samples. While shallow WGS cannot detect copy-neutral loss of heterozygosity or rearrangements, it is cost-effective, with a quality comparable to array CGH and applicable to DNA isolated from the FFPE samples. This allowed us to include samples that had been archived for over 30 years and collate a representative cohort, including LGG patients with long survival.

The relatively low incidence of LGGs and relatively long overall survival of patients necessitated this retrospective, multi-center approach. The observed variability in postoperative treatment can be attributed to the lack of a standard of treatment for these patients. Despite these variations, distal 10q loss (including whole chromosome losses) was significantly associated with an unfavorable prognosis in the discovery, validation and confirmation cohorts. Previously, some studies with smaller cohorts of specific histological subgroups of LGG have reported a correlation between 10q and survival [6,7]. Furthermore, a high prevalence of whole chromosome 10 loss and strong negative correlation with survival have been reported for grade III and IV gliomas [14-17]. Partial loss of 10q is much more frequently detected in histological grade II diffuse gliomas compared with grade III and IV gliomas. In each of the previously published studies the whole of chromosome 10 or the entire 10q arm was taken into account. Here we demonstrate that, different from higher grade gliomas, the distal end of 10q is frequently lost and associates with overall survival in three cohorts. Spatial as well as temporal analyses suggest that subclones with distal loss of 10q are involved in tumor progression, since the loss surfaces in paired recurrent tumors with a higher malignancy grade.

Identification of the genes and their proteins affected by CNAs may elucidate the biological underpinnings of their clinical relevance but can be challenging, especially when a genomic region is large. Only after many years were mutations in *CIC* and *FUBP1* associated with co-deletion of chromosomal arms 1p and 19q [18,19]. In total 148 genes are located on 10q25.2-qter, including *MGMT*, *DMBT1* and *ERCC6* [16,20,21], while the usual suspect, *PTEN*, is located more proximal to the centromere [16] and is preferentially lost in higher grade gliomas [4].

Based on our findings, we suggest that patients with an LGG should be simultaneously tested for both 1p/19q co-deletion and distal loss of 10q, since these two phenomena seem to have counteractive effects on survival. Introduction of heterogeneous CNAs, such as distal 10q loss, in daily clinical practice requires a robust diagnostic test. The well-known clonal features of 1p/19q co-deletion have been helpful to interpret these intermediate copy number levels in LGGs [13]. Analysis of multiple spatially distinct regions could reveal subclones. We currently favor genome-wide analysis, which visualizes chromosomes 1p, distal 10q and 19q simultaneously, and at the same time may provide insight into intratumoral heterogeneity within one region. Both extent of resection as well as the subclonal character of important markers for progression command alternative diagnostic procedures to assess their presence in a postsurgical situation, which may in the future be offered through peripheral blood screening [4]. Meanwhile, detailed registration of the positions of samples from different regions within a tumor obtained during the same surgery may provide more accurate insight into biologically relevant topics such as the physical distance and direction of growth of tumor subclones as well as the overall extent of heterogeneity of a tumor [22].

## Conclusions

Copy number analysis by shallow WGS is a robust approach for archival clinical LGG specimens. For a large proportion of LGG patients, analysis of CNAs with prognostic value may improve personalized timing of therapy. Thereby, loss of distal 10q without 1p/19q co-deletion is indicative for urgent postoperative treatment, while in LGGs without loss of 10q and with 1p/19q co-deletion a wait-and-scan policy should be considered. The subclonal character of whole or distal 10q loss in a subset of samples emphasizes the need for maximal extent of resection, illustrates that single sample diagnostics may be insufficient for LGG and favors future studies on genome-wide analysis of multiple spatially distinct samples to map tumor progression.

## Materials and methods

### Clinical data and sample collection for discovery, validation and confirmation cohorts

Approval for collection of clinical data and FFPE tumor samples for the 98 patients of the discovery cohort was obtained from the institutional review boards of all five Dutch hospitals, namely the Medical Ethical Committee (in Dutch: Medisch-Ethische Toetsingsingscommissie or METc) of the Academical Medical Center (AMC), the METc of the Isala klinieken in Zwolle, the METc of the VU University Medical Center (VUmc) in Amsterdam, the METc of the St Elisabeth Hospital in Tilburg, and the METc of the Arnhem - Nijmegen Region for samples from Radboud University Medical Center in Nijmegen (CMO). Experimental methods in this manuscript are in compliance with the Helsinki Declaration. Inclusion criteria and characteristics of the discovery cohort are summarized in Figure 1 and Table 1. Clinical features of the validation cohort, from a French hospital, can be found in Table 1; materials and methods are presented in more detail by Alentorn *et al*. [5]. For the confirmation cohort, copy number data of 531 lower grade glioma patients from the TCGA database were downloaded on 12 June 2014 via the Cancer Browser at UCSC [23]. Clinical data were available for 373 of these patients, including grade and overall survival; 184 of these samples were categorized as ‘diffuse glioma histological grade 2’ and selected as a confirmation cohort [10]. Presumably as a consequence of the fact that only fresh frozen samples were included in the TCGA cohort, a limited number of patients are contained in the dataset that had deceased during follow-up.

### Laboratory techniques

Histological revision of samples in the discovery cohort was performed by two experienced neuropathologists (EA and PW). For all samples in the discovery cohort, including paired recurrent tumors, areas containing >60% tumor cells were outlined on hematoxylin and eosin stained slides, and tumor cell percentage estimated and registered for each sample (Additional files 2 and 3 and Table S1 in Additional file 4) and 10 adjacent sections were used for DNA isolation [24]. For the assessment of intratumoral heterogeneity, spatially distinct regions were selected based on histological variability and/or plain physical distance (Additional file 4). These samples were obtained either from one FFPE block, or individual blocks from the same surgery (Table S1 in Additional file 4). DNA (500 ng) was fragmented by sonication (Covaris™ S2, Woburn, MA, USA), and sequenced using a 50 bp single-read (50 bp SR) modus (Illumina TruSeq DNA-kit and HiSeq 2000, San Diego, CA, USA). The 100 bp paired-end (100 bp PE) sequencing modus and array CGH were used for comparison and technical validation. Array CGH was performed as described previously [25]. *IDH1* and *IDH2* mutation analysis was performed as described previously [5].

### Statistical analysis

Copy number data from shallow WGS were analyzed using a novel Bioconductor script called QDNAseq [26]. QDNAseq infers copy numbers through depth of coverage by binning reads uniquely aligned to the human reference genome build GRCh37/hg19 with Burrow’s Wheeler Alignment (BWA) [27]. PCR duplicates and reads with mapping qualities below 37 (highest value returned by BWA) were filtered. Copy numbers were inferred from the number of sequence reads per 15 kb bin. A simultaneous Loess correction for sequence mappability and GC content is applied within QDNAseq, which reduces noise of the copy number profiles, particularly for those with more degraded DNA. Problematic genome regions were furthermore filtered by applying our procedures to sequence data from the 1000 Genomes Project [28] to obtain a blacklist that eliminates problematic regions and the most common copy number variants of germ-line origin. Sequence data as well as all array CGH data have been uploaded to the European Genome-phenome Archive (EGA; accession number EGAS00001000643).

Calling of CNAs into discreet categories (normal, gain or loss) for the discovery and validation set was performed with the Bioconductor/R-package CGHcall [12]. A weighted hierarchical clustering of the CNAs was performed using call probabilities to assess similarity of chromosomal profiles [29]. Association with survival was tested using a log rank test with significance estimated over 10,000 permutations. After discovery of regions of interest for survival, consecutive regions in the same chromosome with *P*-values <0.05 were fused together to final regions and the log rank test was repeated. Chromosomal regions that were still significant in the discovery set after multiple testing correction according to Benjamini-Hochberg were verified in the independent French validation cohort. Therefore, genomic coordinates were converted to the NCBI35/hg17 genome build using the UCSC liftOver tool [30] and *P*-values were calculated with the log rank test and adjusted with the more stringent Holm-Bonferroni method. The statistical significance of a CNA was calculated compared to the rest of the cohort not bearing this CNA - for example, samples with loss of distal 10q versus samples without loss of distal 10q.

Regions significant in both the discovery and validation cohorts were tested in the TCGA confirmation cohort for which CNA data were generated with Affymetrix SNP 6.0 arrays (Santa Clara, CA, USA). TCGA level 3 copy number data were publicly available at the time of download and mapped to NCBI36/hg18. These level 3 data involve beginning and end positions of chromosomal segments with deflection values, resulting from TCGA preprocessing (for level definitions and preprocessing see [31]). Segment values were converted to CNA discreet categories by setting thresholds whereby a log2 ratio of >0.20 is gain, < -0.23 is loss and all other values are normal copy number; these values correspond to 30% of the tumor cells with that CNA. Those segments overlapping for at least 90% of the 1p, 19q (excluding centromeres) or 10q25.2-qter region (corresponding to NCBI35/hg17: chr10: 112939991-135323881 and NCBI36/hg18: chr10:112939991-135284990) were taken into consideration. At this setting 14 patients had a distal 10q loss, of which 4 deceased during follow-up and no patients had both distal 10q and 1p/19q loss. *P*-value calculations were performed as described above without corrections since only one region was tested for confirmation. The threshold settings were selected based on the fact that a deletion in 30% of the tumor cells, as observed for the chromosome 10 loss in LGG sample 240 (Figure S3 in Additional file 1), should not be missed. All other threshold values for calling losses of these regions were stepwise tested as well as the percentage of overlap with the 10q25.2-qter region and are presented in Table S2 in Additional file 4. Significance for the 10q loss remained for many different settings, but the number of patients with this loss substantially decreased with widening margins for calling CNAs to lower than should be expected based on the discovery and validation cohorts.

To assess the presence of CNAs between spatially distinct regions and/or recurrences from the same patient, common regions were detected with CGHregions [32], and regions smaller than 5 Mbp were excluded. Clinical parameters were analyzed with log rank test. All reported *P*-values were two-sided, and <0.05 was considered statistically significant.

### Data availability

Both array CGH and sequence data have been uploaded to the European Genome-phenome Archive (EGA; accession number EGAS00001000643).

## Additional files

Additional file 1: Figure S1. Evaluation of shallow WGS for genome-wide copy number analysis for read lengths of sample LGG 284. Copy number profiles were produced by counting the number of uniquely mapped sequence tags per 15 kb bin for different settings. **(A)** Paired-end 100 (PE100), 100 bp reads from both ends. **(B)** Single-end 100 (SR100), 100 bp reads. **(C)** Single-end 50 (SR50), 50 bp reads from one end. **(D)** Technical validation by array CGH of the same sample. Y-axis, normalized log 2 sequence read counts per bin; x-axis, 15 kb bins ordered by genomic position from chromosomes 1 to22. **Figure S2.** Technical validation of shallow WGS by array CGH CNA analysis of eight LGGs tested on both platforms. The level of deflection of CNAs was comparable. Also, heterogeneously present CNAs with only marginal deflection could be reproduced; LGGs 168, 184 and 193 show clonal distal 10q loss, while LGGs 187, 189, 210, 211 and 224 show subclonal loss of 10q. **Figure S3.** Minimal tumor cell percentage required for detection of CNAs. Calculation of the percentage of tumor cells with a chromosome 10 deletion in LGG240. To simulate a limited tumor percentage, a virtual tumor-normal mixture experiment was performed with LGG240 (diffuse low-grade glioma) and NA18960 ((1000 Genomes Project Consortium). **Figure S4.** Technical validation of shallow WGS by array CGH. Technical validation of LGG240 (original) and three spatially distinct regions. Additional file 2:
**Copy number profiles generated with shallow WGS of original LGGs of the discovery cohort and spatially distinct regions of this tumor obtained during the same surgery.** A representative picture of histology (hematoxylin and eosin staining, original magnification × 200) is depicted in the top left corner of each profile. Additional file 3:
**Copy number profiles generated with shallow WGS of initial LGGs and paired recurrent tumors.** A representative picture of histology (hematoxylin and eosin staining, original magnification × 200) is depicted in the top left corner of each profile. Additional file 4: Table S1. Overview of all samples of the discovery cohort, including paired recurrent tumors and spatially distinct regions. Columns show ID number, relative spatial distance (one FFPE block or individual FFPE block), histology, prognostically relevant CNAs, distal 10q loss, 1p/19q co-deletion, read count, sequence depth and tumor cell percentage as estimated by neuropathologists. **Table S2A-C.** Matrix with *P*-values for overall survival of the TCGA LGG (grade 2) confirmation cohort at various thresholds. Vertical rows: log2 ratio thresholds for calling CNA discrete categories (loss, normal or gain) from the log2 ratio level 3 data downloaded from the Cancer Browser at UCSC [23]. Horizontal rows: thresholds for overlap with the 10q25.2-qter region. Grey, non-significant settings; shades of beige, significant settings (darker indicates higher signioficance); bold, threshold settings used for Figure 3. (A) *P*-values rounded to four decimal places for samples with or without a distal chromosome 10q loss. (B) Number of grade 2 LGG patients with clinical data (n =184) from the lower grade glioma TCGA dataset with a distal 10q deletion. (C) Number of grade 2 LGG patients (n =184) from the lower grade glioma TCGA dataset with a distal 10q deletion that had deceased during follow-up. Overall survival is significant for various settings of distal 10q loss. The number of patients with distal 10q loss substantially decreased with widening margins for calling CNAs, to lower than should be expected based on the French and Dutch cohorts.

## Abbreviations

bp: base pair
CGH: comparative genomic hybridization
CNA: copy number aberration
FFPE: formalin-fixed paraffin-embedded
LGG: diffuse low-grade glioma
PCR: polymerase chain reaction
TCGA: The Cancer Genome Atlas
WGS: whole genome sequencing
