# Supplementary material for: Spatial and temporal evolution of distal 10q deletion, a prognostically unfavorable event in diffuse low-grade gliomas
Source: Genome Biol. 2014 Sep 23;15(9):471. doi: 10.1186/s13059-014-0471-6 (PMC4195855; doi:10.1186/s13059-014-0471-6)

Figure S1

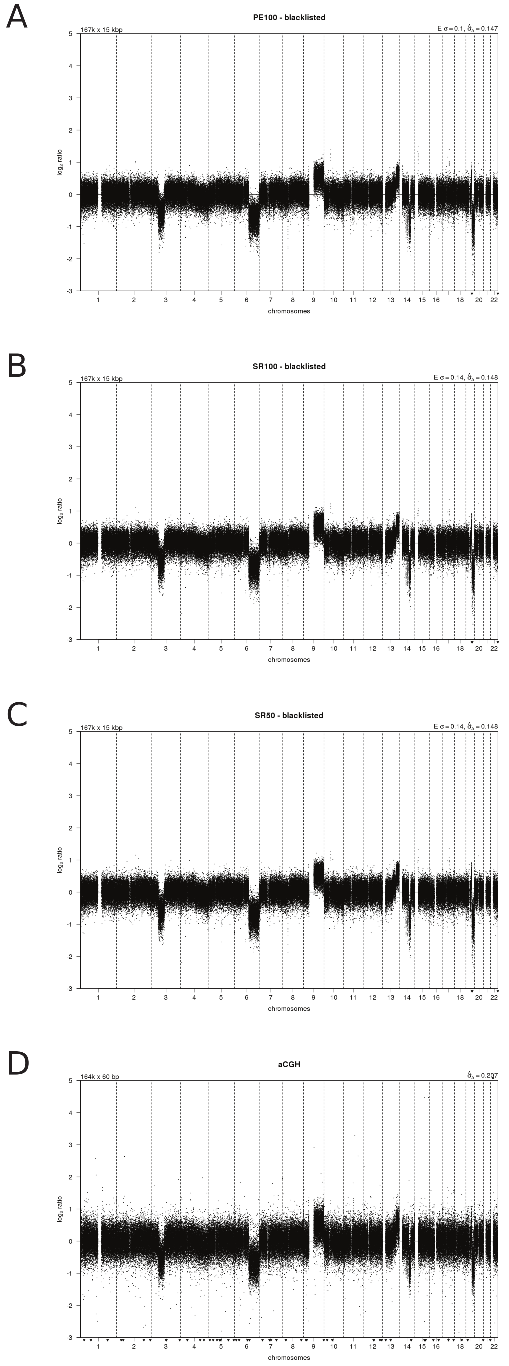

Figure S1. The standard deviation of a profile, denoted by Sigma hat Delta, and the theoretically expected standard deviation based on read counting, denoted by E Sigma, are given in the top right corner of each profile. In the top left corner the number and size of bins used for counting of reads are indicated.

Figure S2

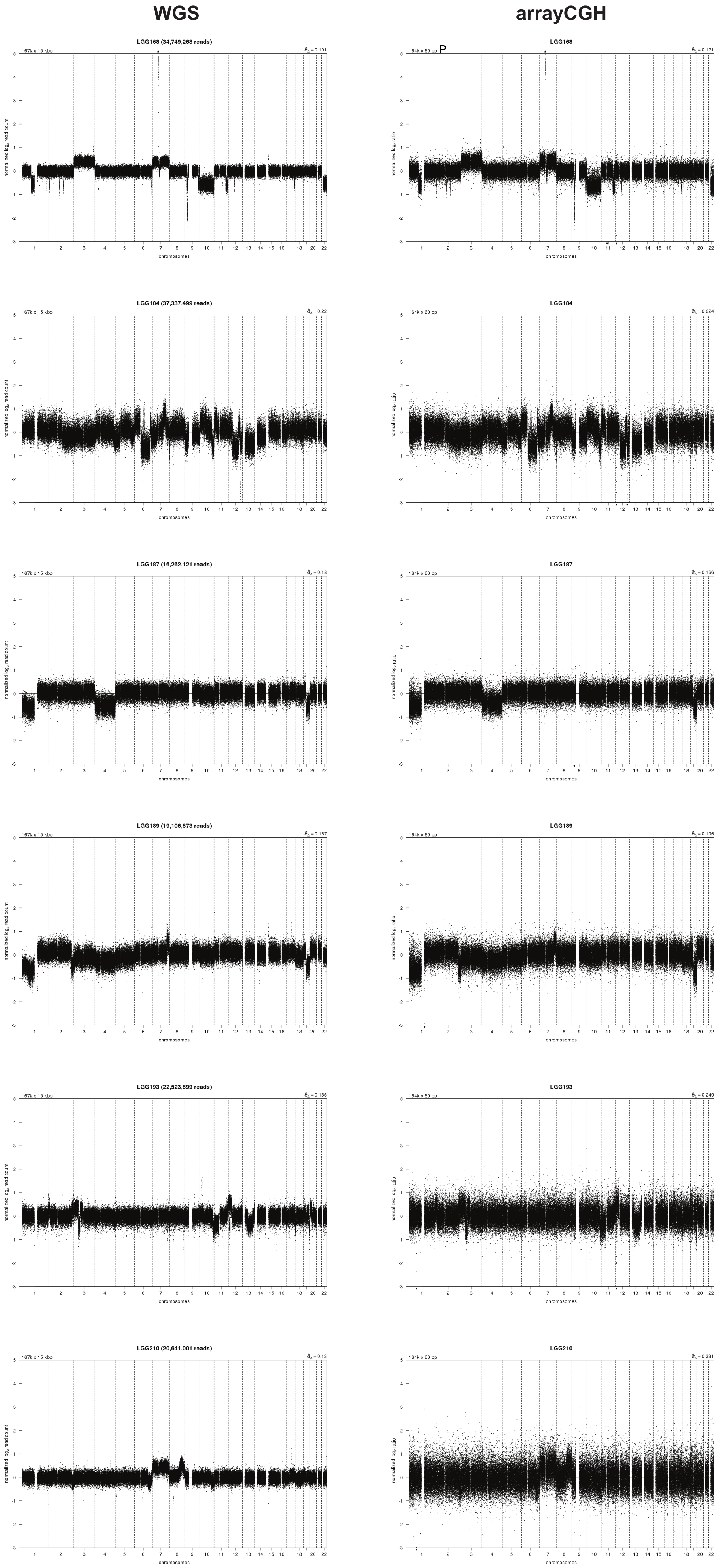
WGS
arrayCGH
LGG168 (34,749,268 reads)
LGG168
LGG184 (37,337,499 reads)
LGG184
LGG187 (16,262,121 reads)
LGG187
LGG189 (19,106,673 reads)
LGG189
LGG193 (22,523,899 reads)
LGG193
LGG210 (20,641,001 reads)
LGG210
167k x 15 kbp
164k x 60 bp
normalized log2 read count
normalized log2 ratio
chromosomes

WGS

arrayCGH

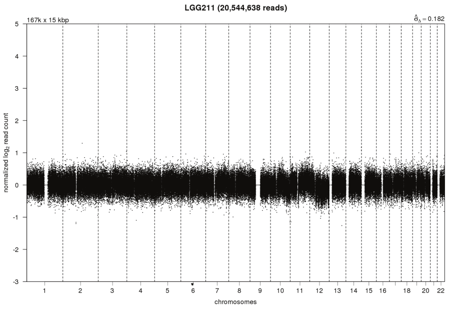

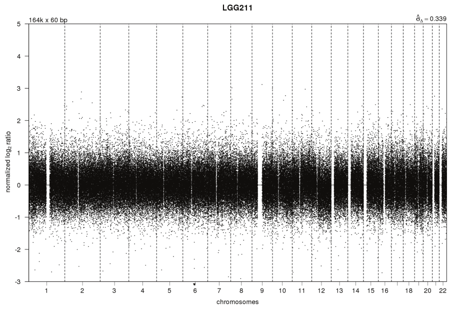

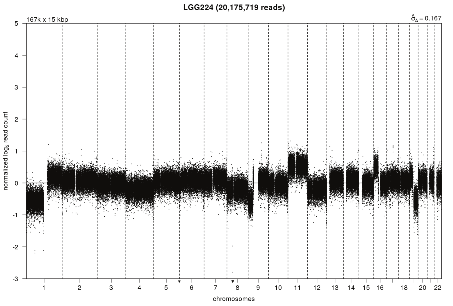

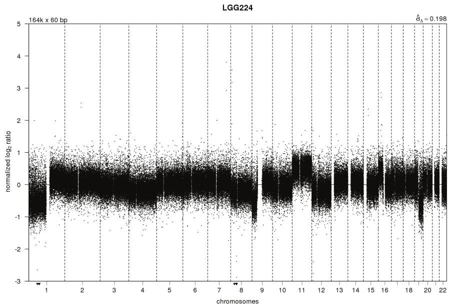

Figure S3

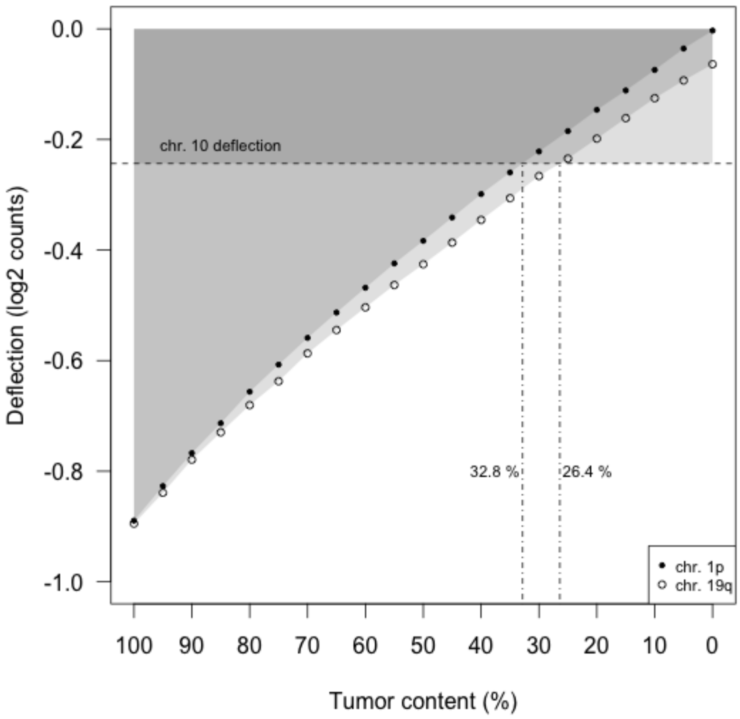

Figure S3. A mixture series, ranging from 100% to 0% with steps of 5% (x-axis), is performed while maintaining a total of 10 million reads for each mixture. The deflection of chromosome 1p and 19q deletion (y-axis) decreases with lower tumor content and intersects with the level of chromosome 10 at respectively 32.8% and 26.4% tumor content. Given the high repeat and GC content of chromosome 19, the intersection with chromosome 1p (32.8%) is considered a more realistic estimate for the percentage of tumor cells containing the chromosome 10 deletion.

Figure S4

WGS **arrayCGH**

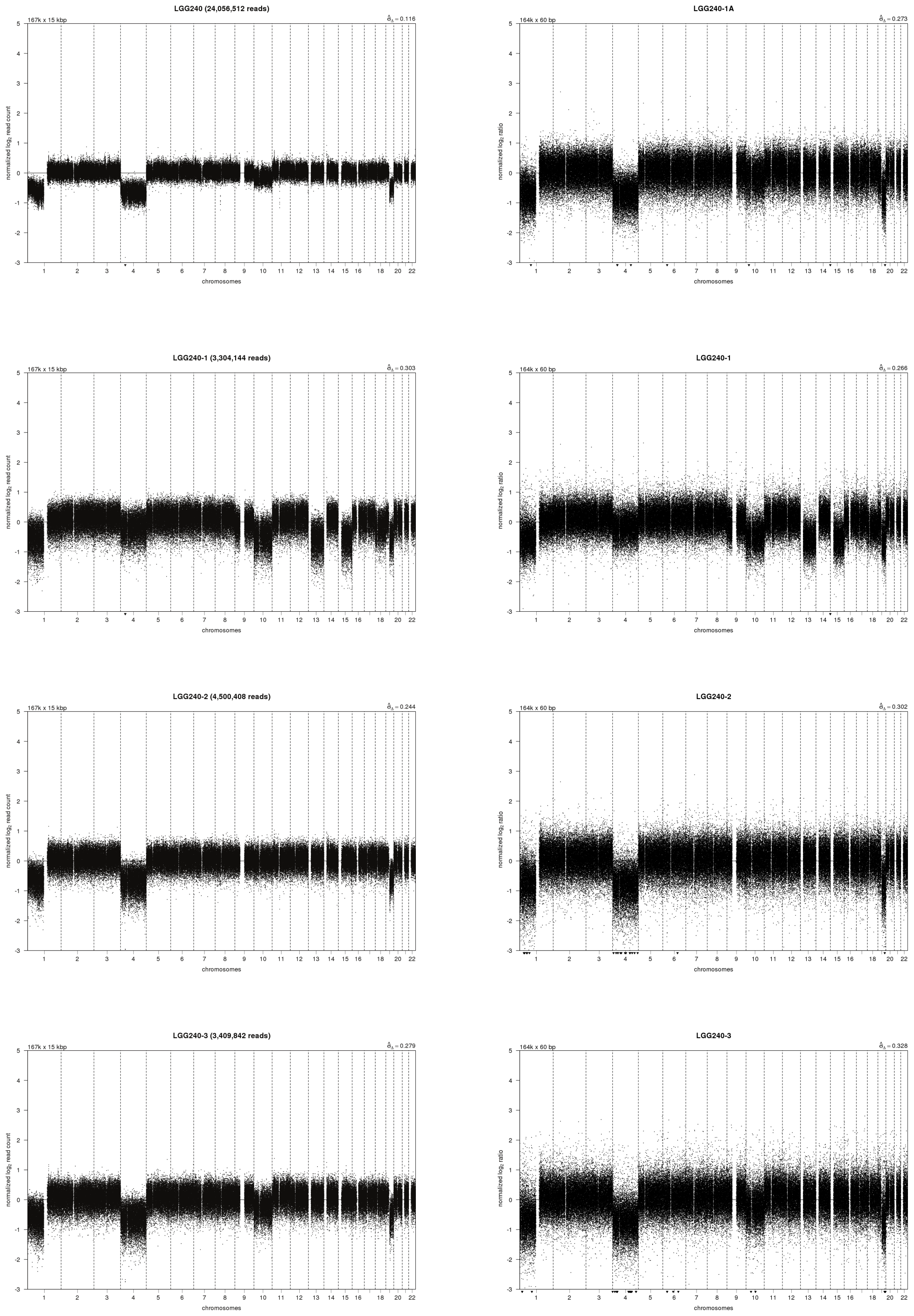

Supplement: Additional file 1: Figure S1. — Evaluation of shallow WGS for genome-wide copy number analysis for read lengths of sample LGG 284. Copy number profiles were produced by counting the number of uniquely mapped sequence tags per 15 kb bin for different settings. (A) Paired-end 100 (PE100), 100 bp reads from both ends. (B) Single-end 100 (SR100), 100 bp reads. (C) Single-end 50 (SR50), 50 bp reads from one end. (D) Technical validation by array CGH of the same sample. Y-axis, normalized log 2 sequence read counts per bin; x-axis, 15 kb bins ordered by genomic position from chromosomes 1 to22. Figure S2. Technical validation of shallow WGS by array CGH CNA analysis of eight LGGs tested on both platforms. The level of deflection of CNAs was comparable. Also, heterogeneously present CNAs with only marginal deflection could be reproduced; LGGs 168, 184 and 193 show clonal distal 10q loss, while LGGs 187, 189, 210, 211 and 224 show subclonal loss of 10q. Figure S3. Minimal tumor cell percentage required for detection of CNAs. Calculation of the percentage of tumor cells with a chromosome 10 deletion in LGG240. To simulate a limited tumor percentage, a virtual tumor-normal mixture experiment was performed with LGG240 (diffuse low-grade glioma) and NA18960 ((1000 Genomes Project Consortium). Figure S4. Technical validation of shallow WGS by array CGH. Technical validation of LGG240 (original) and three spatially distinct regions. [file 13059_2014_471_MOESM1_ESM.pdf]
