# Supplementary figures and images for: Spatial and temporal evolution of distal 10q deletion, a prognostically unfavorable event in diffuse low-grade gliomas
Source: Genome Biol. 2014 Sep 23;15(9):471. doi: 10.1186/s13059-014-0471-6 (PMC4195855; doi:10.1186/s13059-014-0471-6)

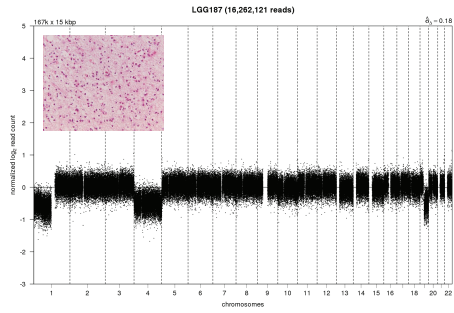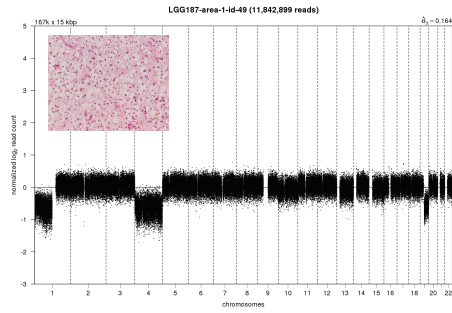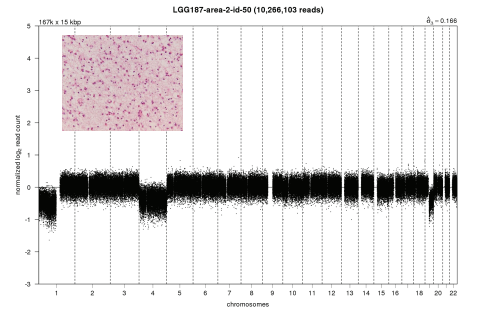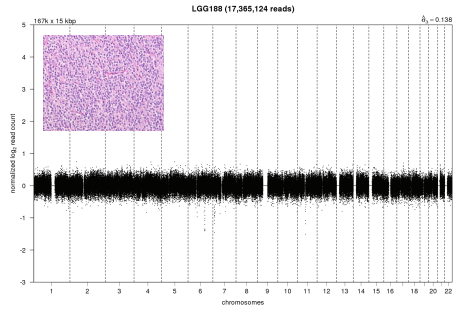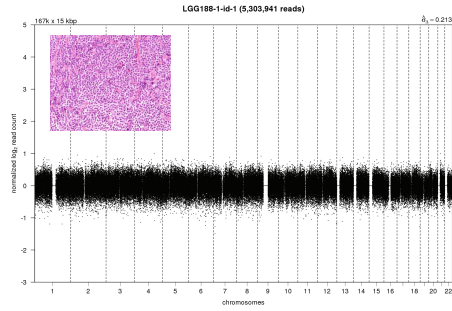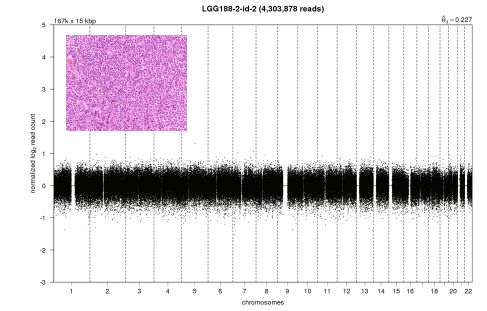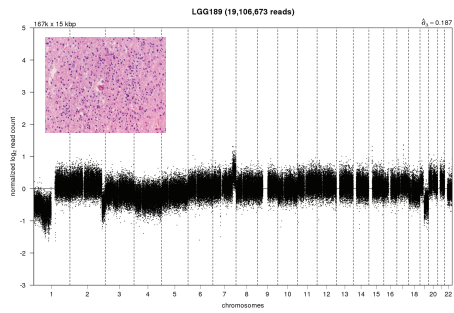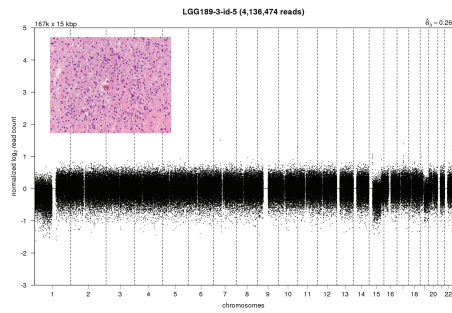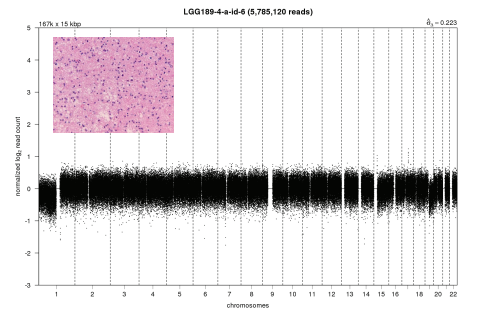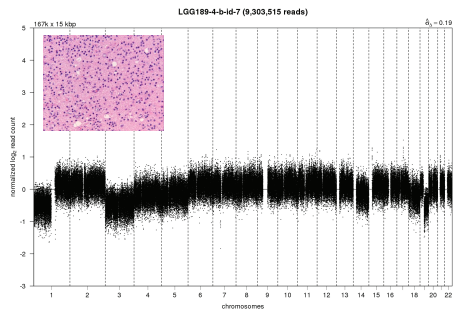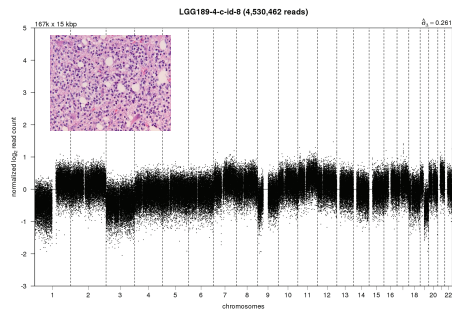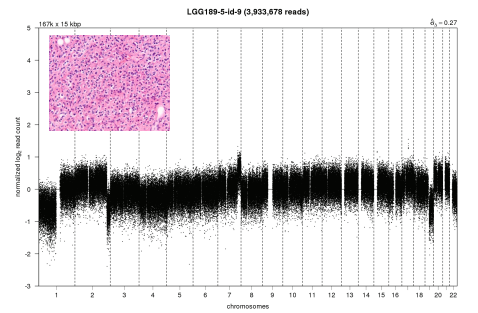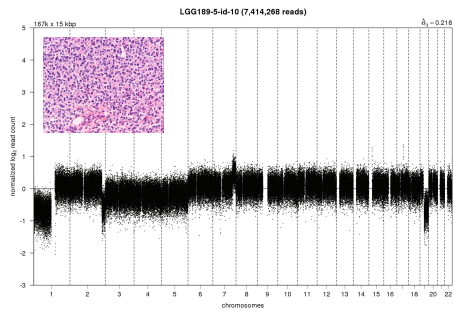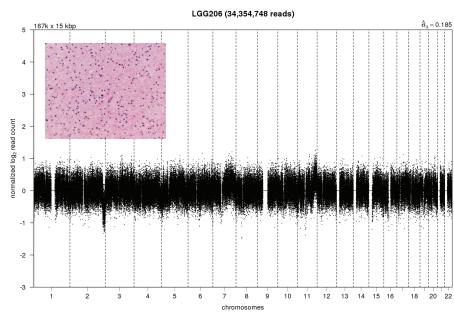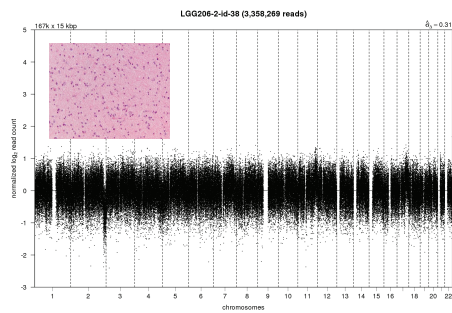

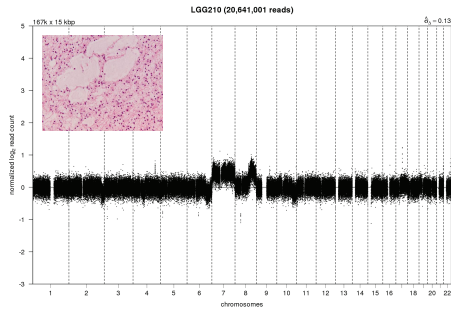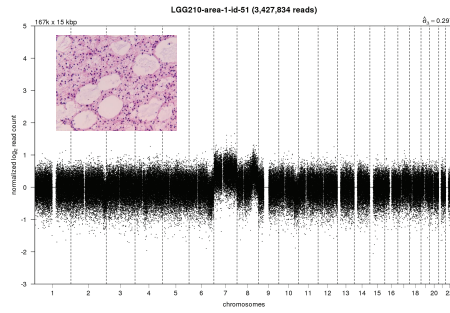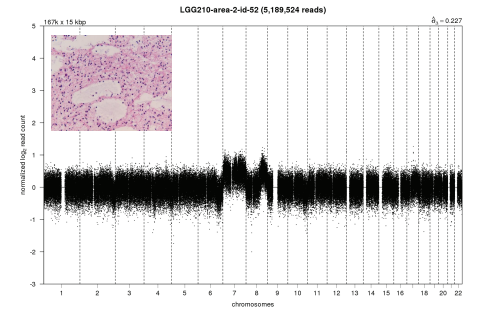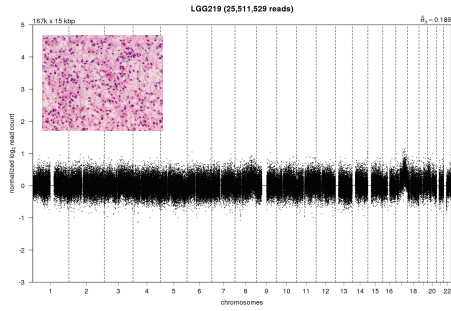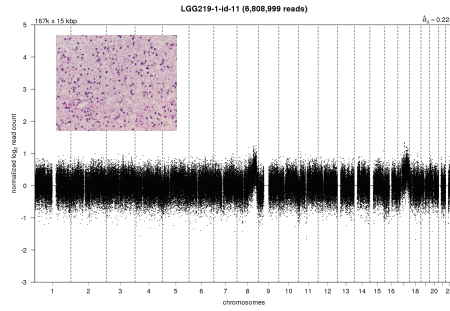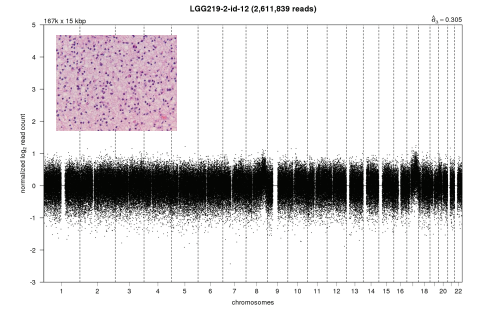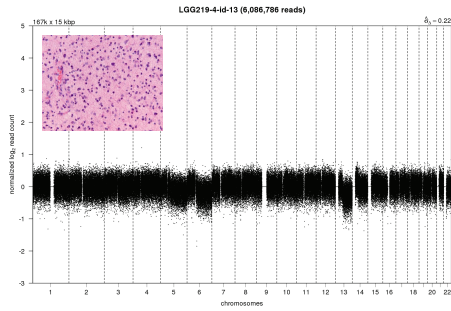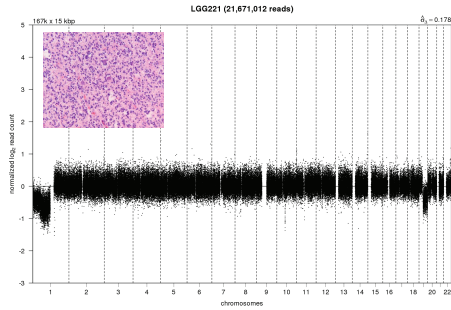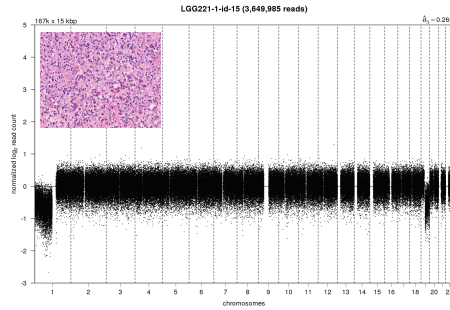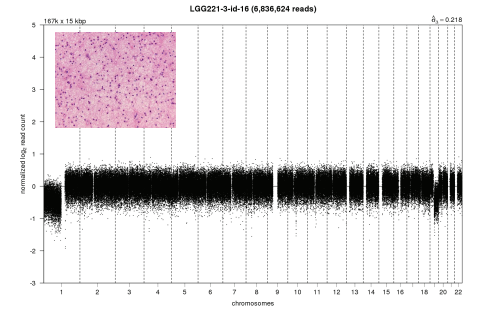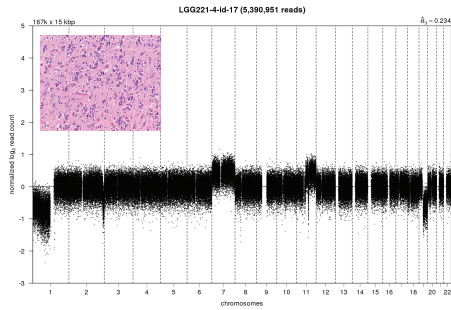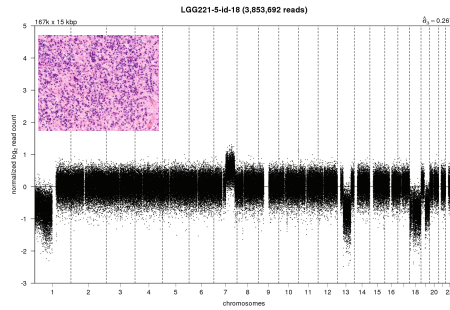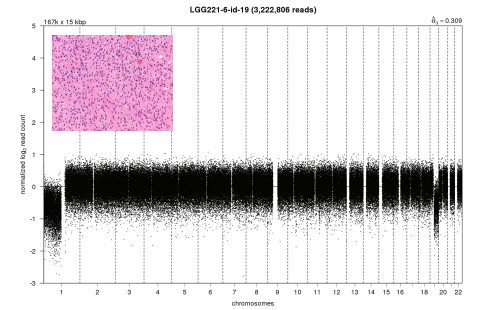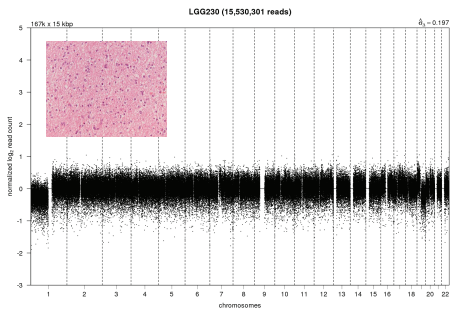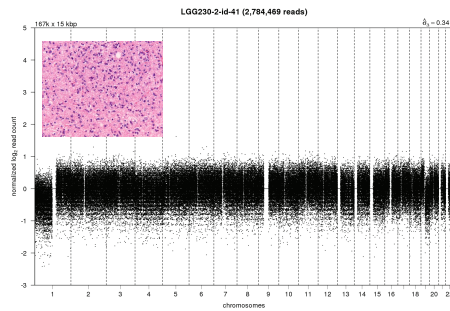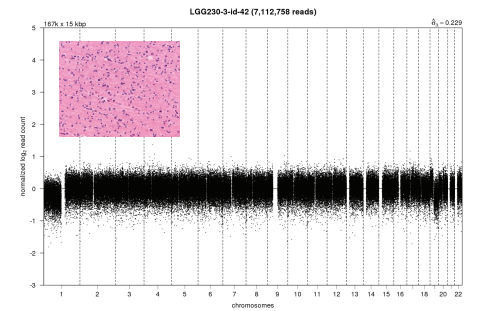

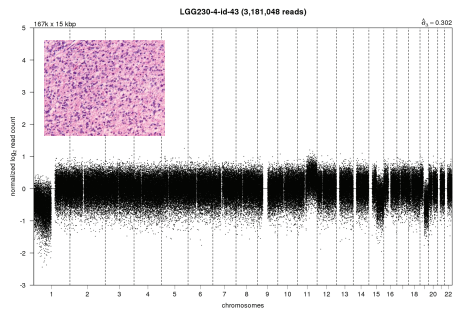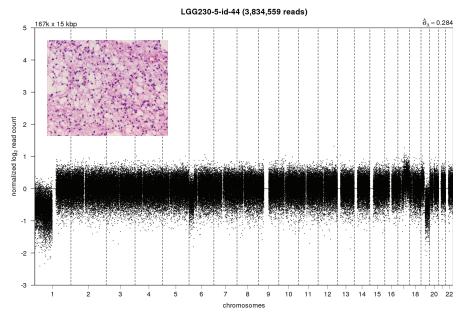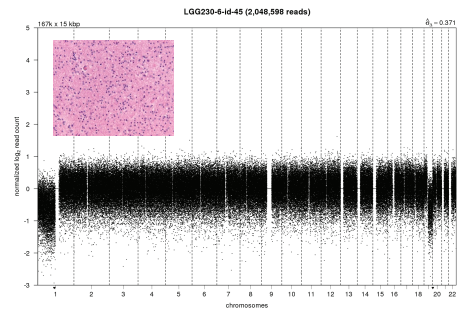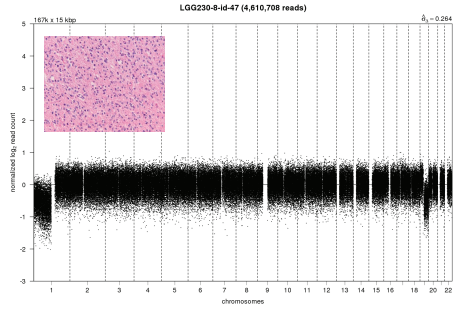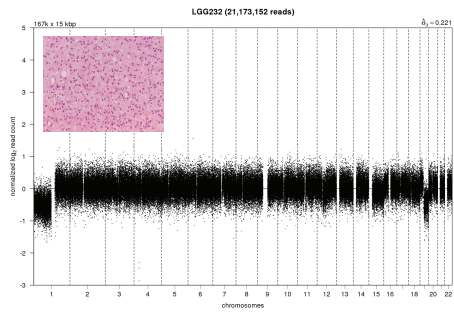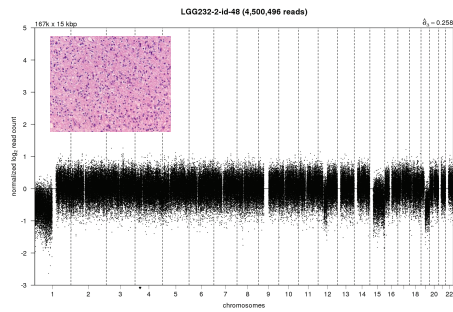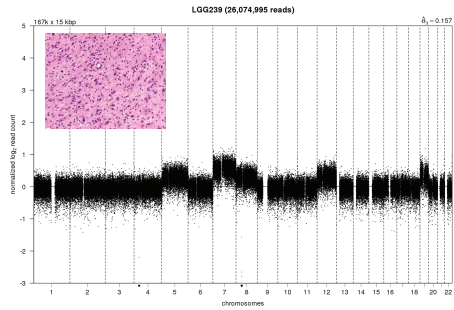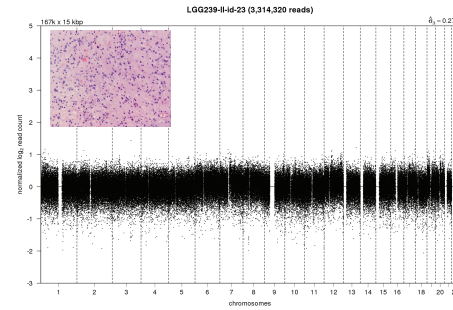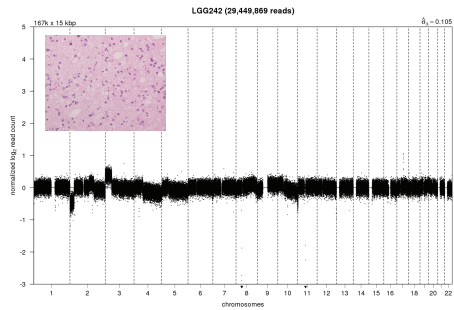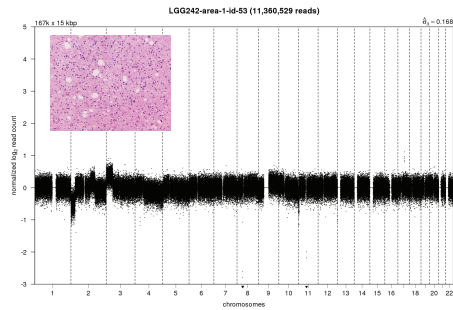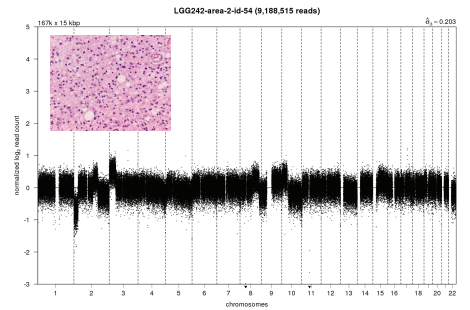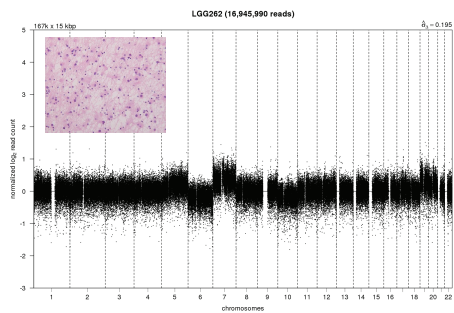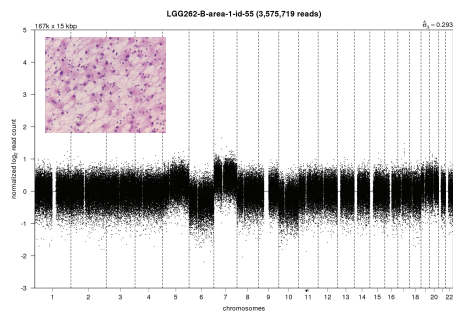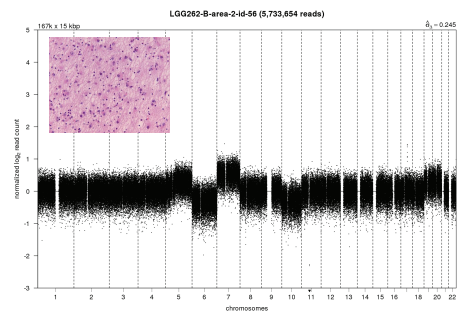

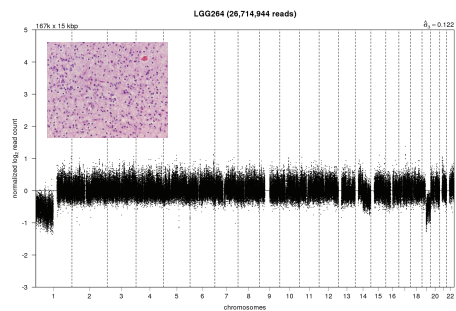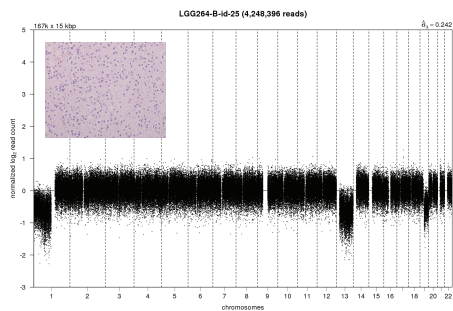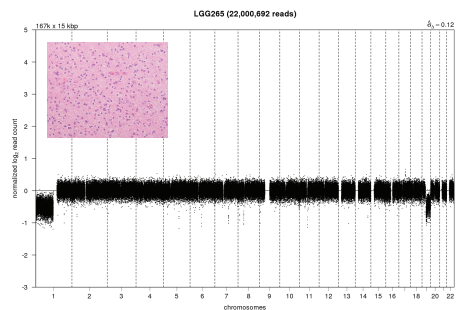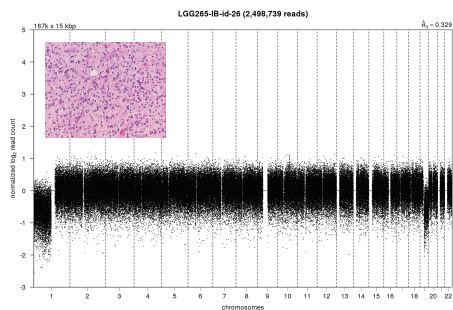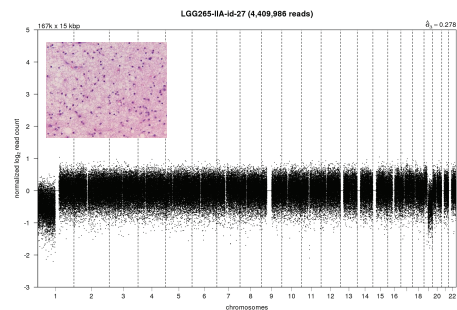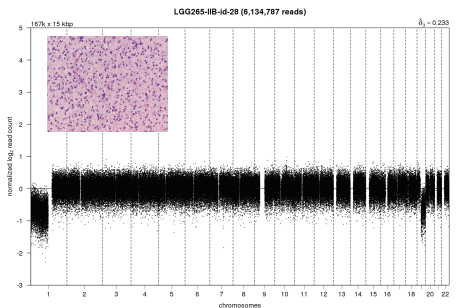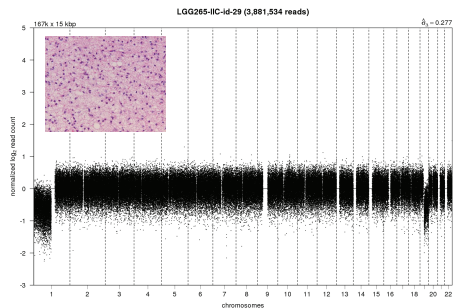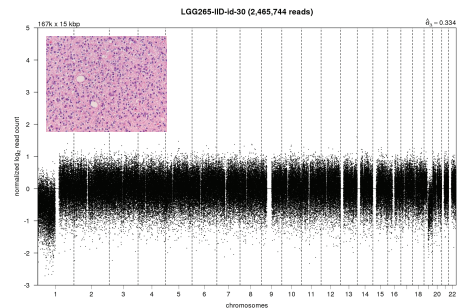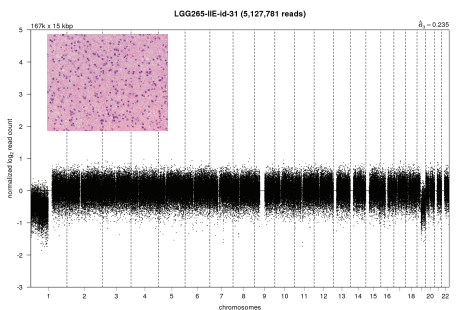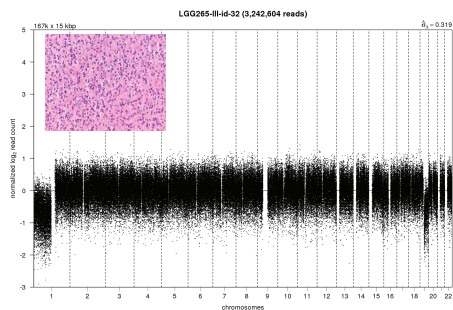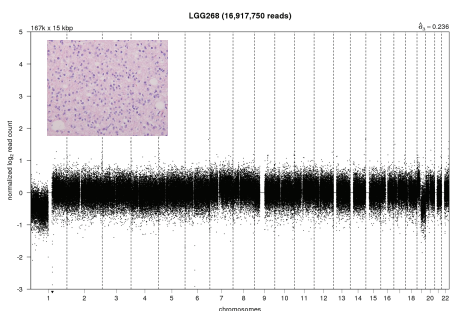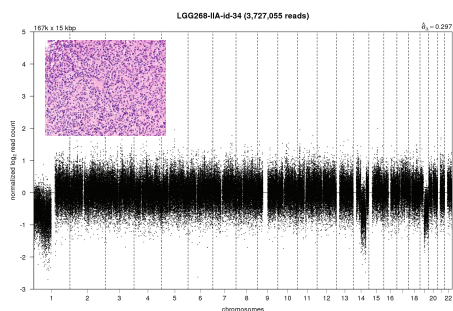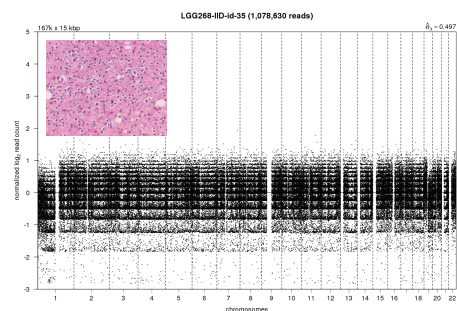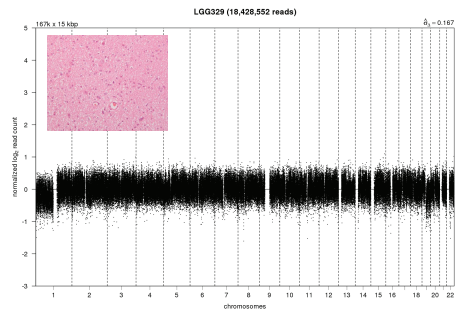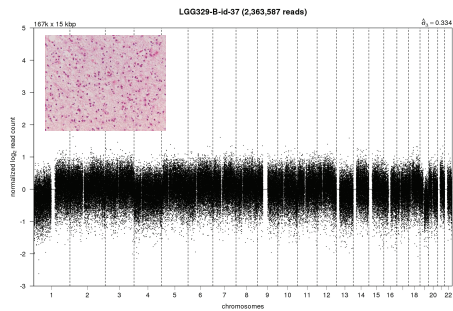

Supplement: Additional file 2: — Copy number profiles generated with shallow WGS of original LGGs of the discovery cohort and spatially distinct regions of this tumor obtained during the same surgery. A representative picture of histology (hematoxylin and eosin staining, original magnification × 200) is depicted in the top left corner of each profile. [file 13059_2014_471_MOESM2_ESM.pdf]

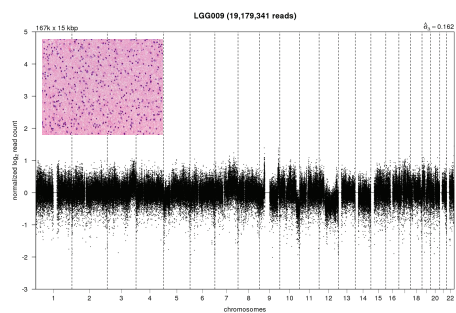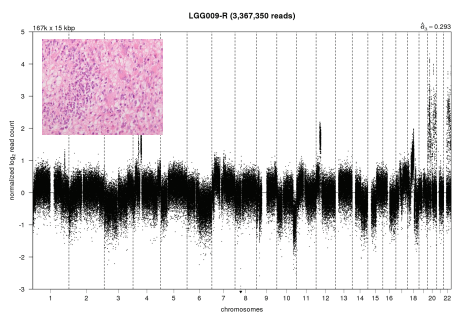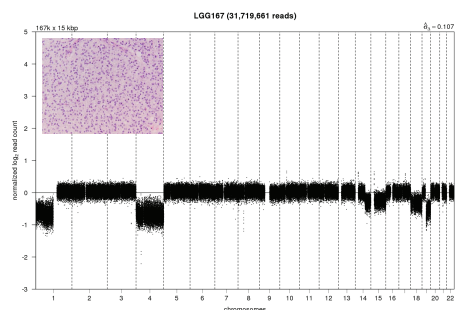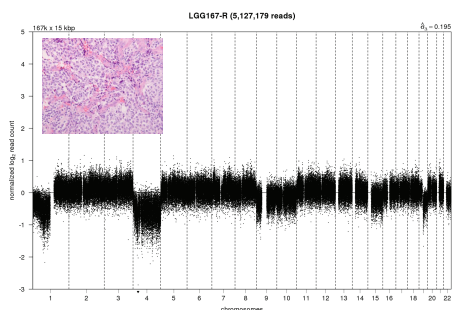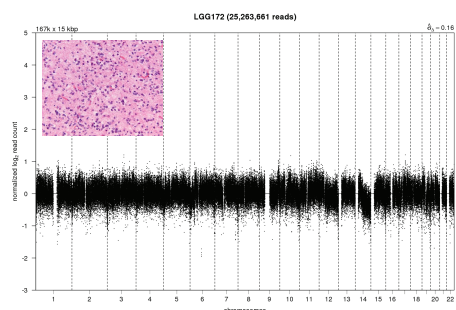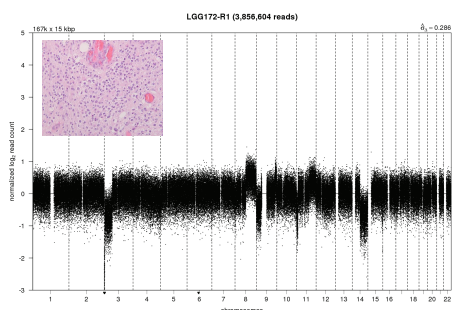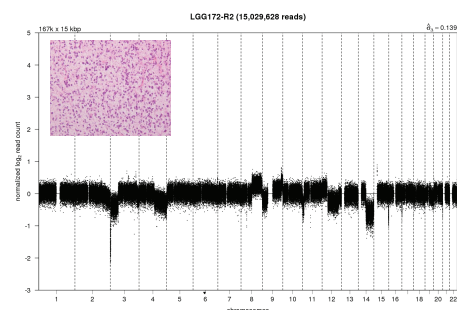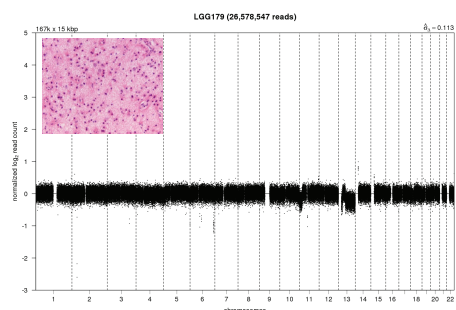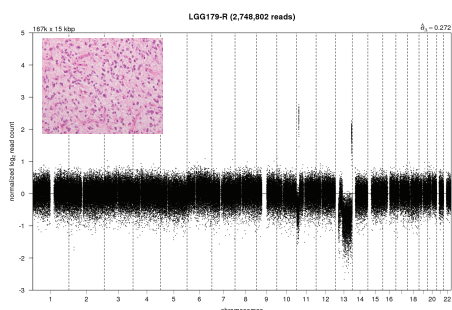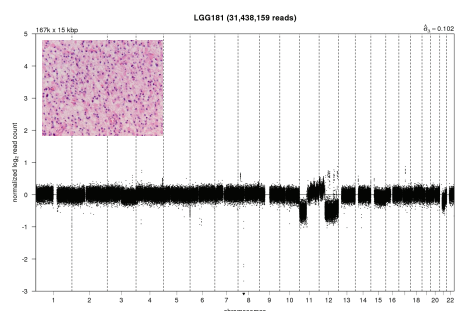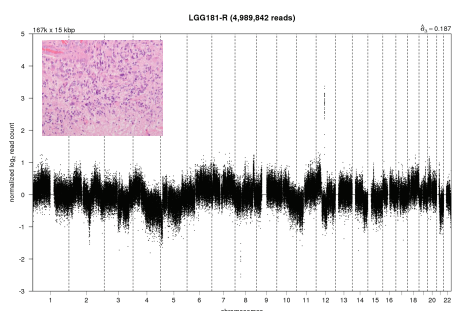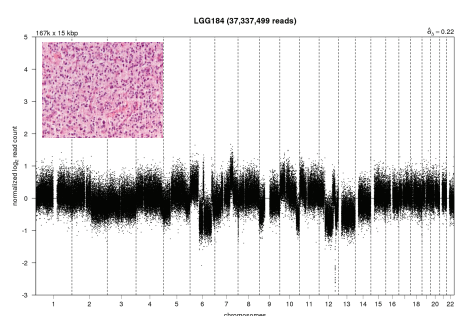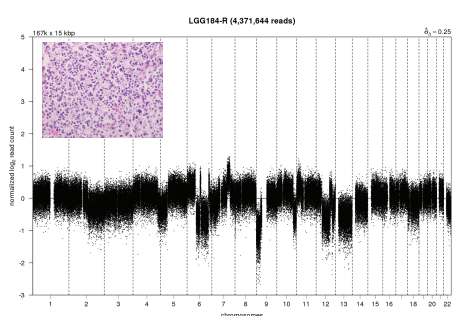

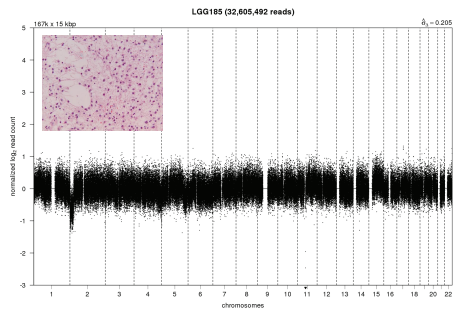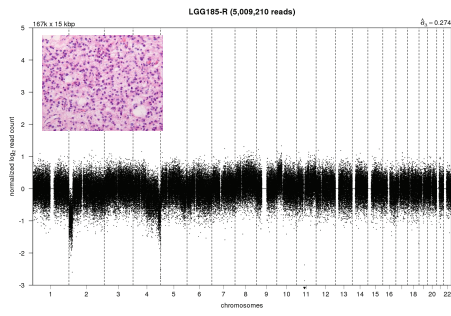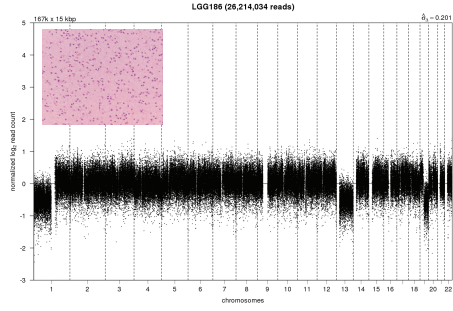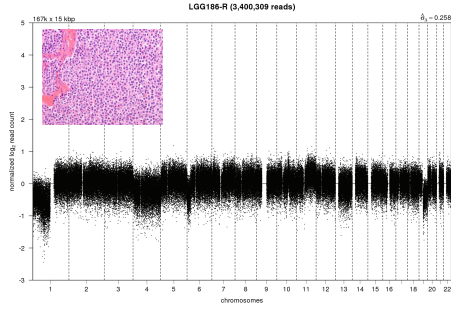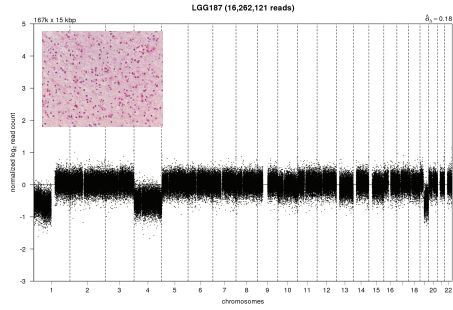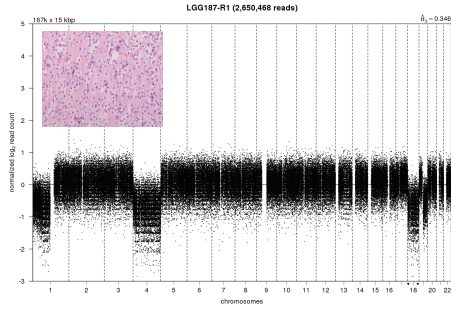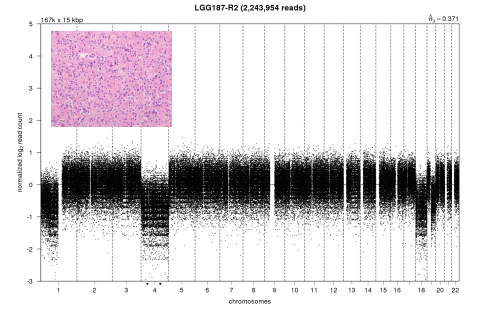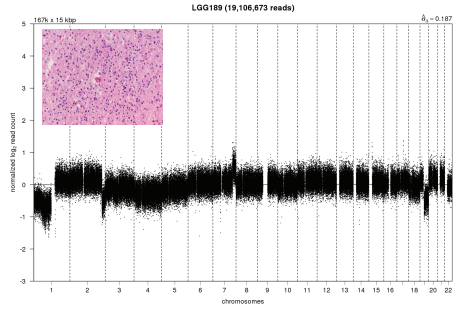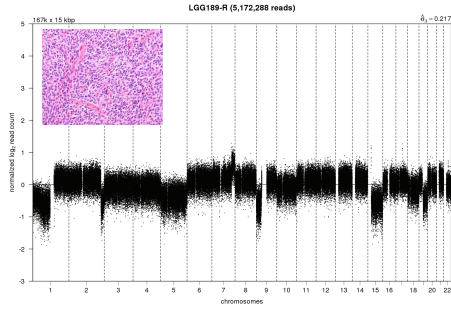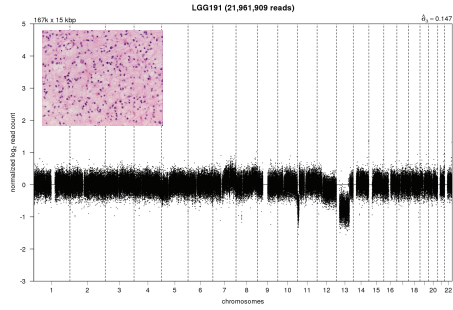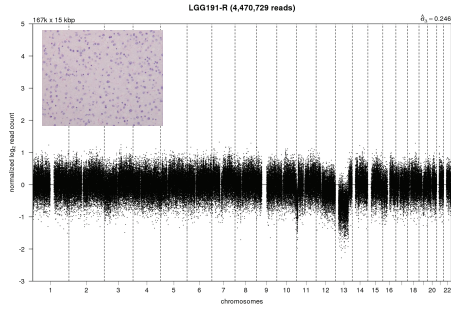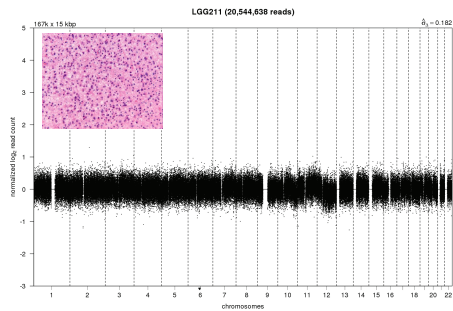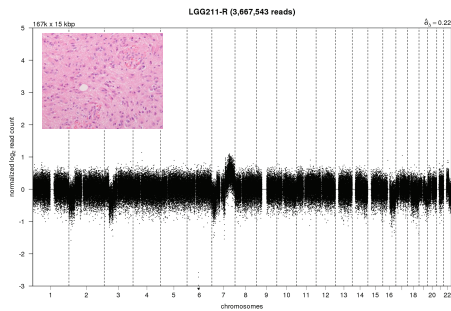

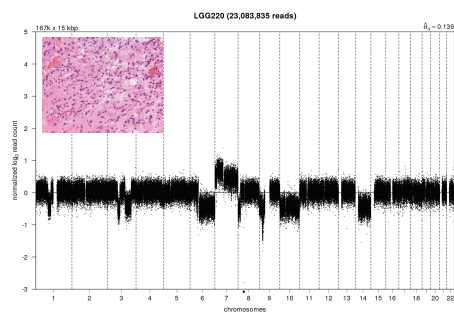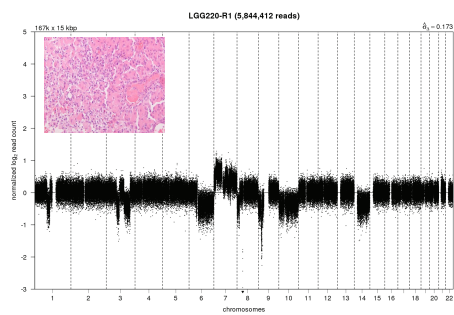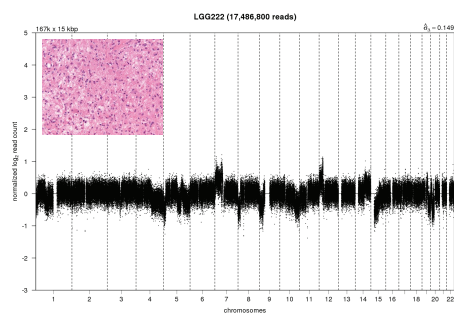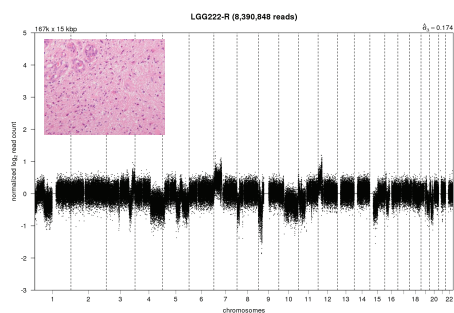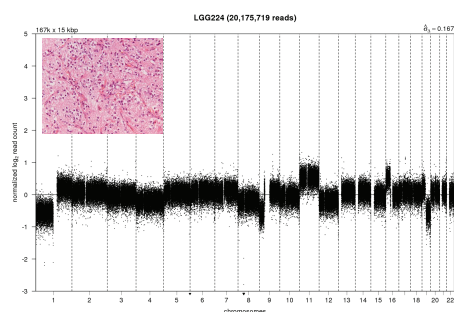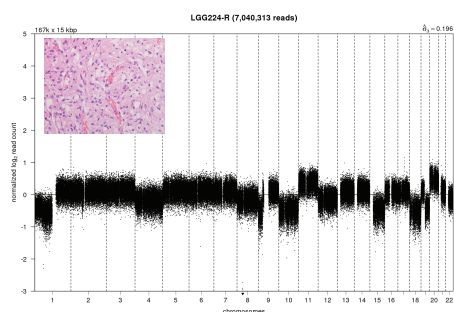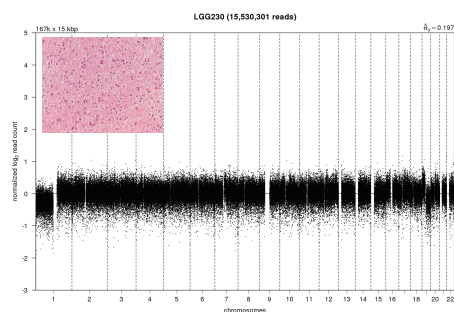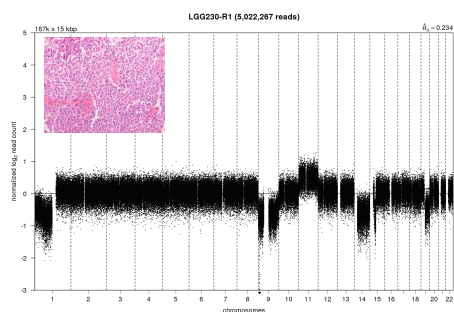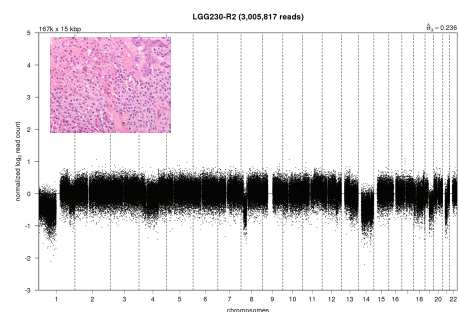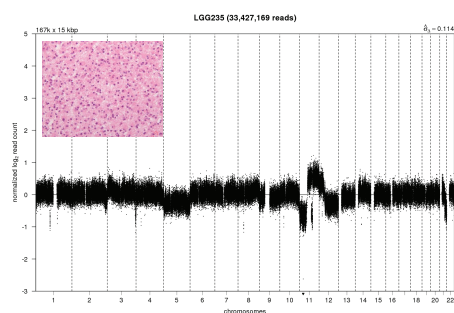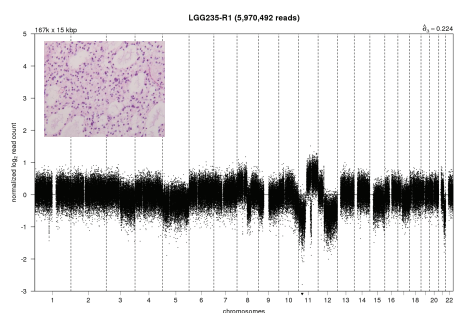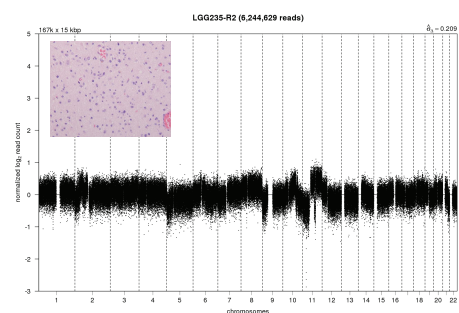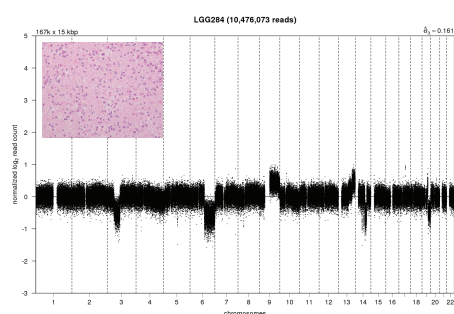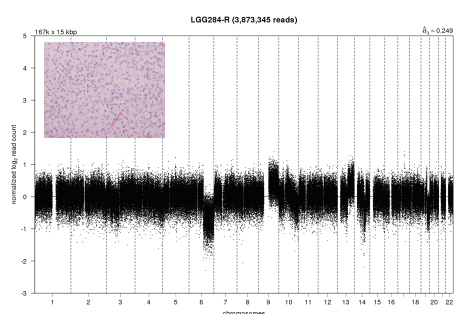

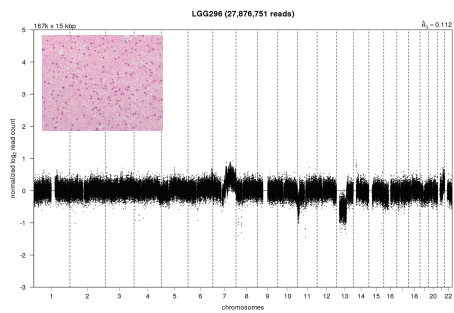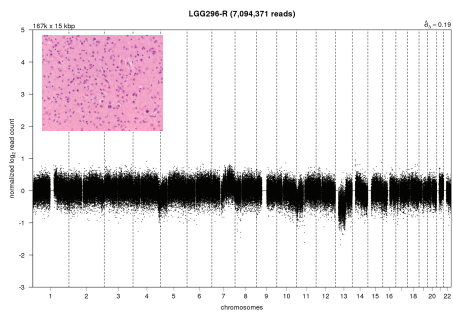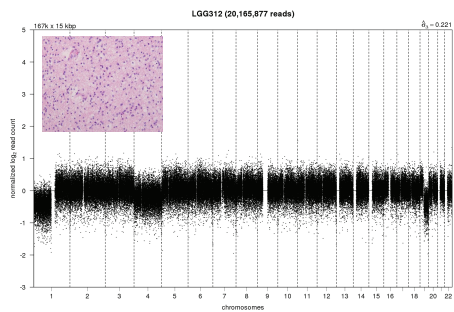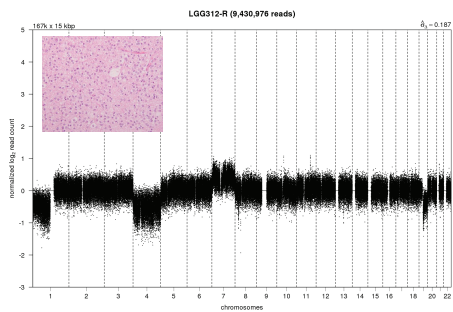

Supplement: Additional file 3: — Copy number profiles generated with shallow WGS of initial LGGs and paired recurrent tumors. A representative picture of histology (hematoxylin and eosin staining, original magnification × 200) is depicted in the top left corner of each profile. [file 13059_2014_471_MOESM3_ESM.pdf]
